# Protecting bacteriophages under UV irradiation with brilliant blue FCF for targeted bacterial control

**DOI:** 10.1016/j.bioflm.2025.100286

**Published:** 2025-05-09

**Authors:** Mateusz Wdowiak, Aneta Magiera, Magdalena Tomczyńska, Witold Adamkiewicz, Francesco Stellacci, Jan Paczesny

**Affiliations:** a Institute of Physical Chemistry, Polish Academy of Sciences, Marcina Kasprzaka 44/52, 01-224, Warsaw, Poland; b École Polytechnique Fédérale de Lausanne, Station 12, CH-1015, Lausanne, Switzerland

**Keywords:** Bacteriophages, Food dyes, Stabilization, Antimicrobial combinations, Food preservation, Biofilms, Membranes

## Abstract

Compared to the standard methods for treating bacterial diseases, bacteriophages are eco-friendly and chemical-free. Exposure to ultraviolet (UV) light or sunlight hampers the efficacy of phage-based approaches. This is crucial when phages are *i)* exposed to sunlight (e.g., in agriculture) or *ii*) are to be used simultaneously with UV for sterilization. Here, we develop a method utilizing a food dye, brilliant blue FCF (BB), that selectively stabilizes bacteriophages against exposure to UV irradiation. In the absence of BB, all tested phages and bacteria are completely inactivated by UV exposure. However, with the addition of BB, all tested non-enveloped phages are effectively protected, while gram-negative bacteria remain vulnerable to UV inactivation. The mechanism of protection requires selective binding of BB to the virion. The simultaneous action of BB-stabilized bacteriophages and UV allows for the removal of up to 99.99 % of bacteria within only 30–60 min. We demonstrate the method's applicability in combating biofouling of membranes and food sterilization. We envision using the developed approach against biofouling in industrial processes, agriculture, and the food industry.

## Highlights

• Selected food dyes show UV-protective properties towards bacteriophages.
• Among tested colorants, only brilliant blue FCF is specific to bacteriophages, not bacteria.
• The specific UV stabilization relies on the interactions of sulfonic compounds with virions.
• The selectivity of brilliant blue FCF is a unique feature among sulfonic compounds.
• The method can be used for biofilm control, industrial sterilization, and food preservation.

## 1 INTRODUCTION

Approximately 13 % of deaths are related to bacterial diseases [1]. Bacteria are also involved in developing some types of cancers [2] and metabolic disorders [3]. Microbial contamination is a leading cause of food spoilage, accounting for the loss of approximately 25 % of food produced annually [4]. Biofouling is one of the biggest threats to the operation of membrane bioreactors, decreasing efficiency and increasing costs. Standard methods for treating bacterial infections, contaminations, and biofilms consist of antibiotics and pesticides; the problem is that both accumulate in the environment and can be potentially harmful to living creatures [5]. Moreover, spreading the antibiotics into the environment would undoubtedly result in the wildfire of antibiotic resistance [6]. Therefore, there is a pressing need to find biocompatible and harmless bactericides, such as bacteriophages.

Bacteriophages are a group of viruses infecting and killing only specific bacterial cells [7] while being generally accepted as safe for eukaryotic organisms, including humans [8,9]. Phages can interact with eukaryotes directly by affecting cells and tissues or indirectly by altering the microbiome [10], and there is minimal evidence of harm during phage treatment [11]. Phages are becoming of interest for various applications, including phage therapy [12], food preservation [13,14], plant protection in agriculture [15], and industrial sterilization [16]. Phages are cheap, easy to produce, safe, specific, and ubiquitous, making them auspicious candidates for future industrial applications. Being on the verge of the nanoscale, some even consider phages as biological, antibacterial nanomachines [17]. The major weakness is their susceptibility to adverse environmental conditions such as elevated temperatures, high pressures, osmotic shock, electric fields, and radiation of different spectrum ranges [18].

The most frequently used antimicrobial agent is ultraviolet (UV) radiation (λ = 100–400 nm). It is efficient in the eradication of bacteria. However, it also affects phage virions by directly generating radicals and damaging the viral genome, causing a decrease in viral infectivity [19,20] or by generating hydroxyl radicals that inactivate viral polymerases [21]. Hence, applying phages exposed to UV (either by choice, where both factors are used to simultaneously fight bacteria, or as an unwanted side effect, e.g., in agriculture) is self-limiting. Our previous study showed the efficacy of Congo red (CR) dye for selective bacteriophage protection from UV irradiation [22]. However, that dye was considered hazardous and potentially harmful to the environment and animals [23]. Therefore, the application of Congo red-based protocols turned out to be limited to procedures that do not involve living creatures, with the necessity of dye recovery from aqueous solutions. Thus, finding protective, selective, and non-hazardous agents seemed indispensable. Several food dyes – including brilliant blue FCF (E132), Allura red (E129), and sunset yellow FCF (E110), are known for their interactions with various proteins [[24], [25], [26], [27], [28], [29]] or even bacteriophages [30,31]. Brilliant blue FCF resembles brilliant blue R-250 (Coomassie blue) used for protein staining in gel electrophoresis. Due to these features, food dyes appear compelling candidates for bacteriophage protection against UV irradiation to facilitate simultaneous sterilization by both factors.

In this study, we develop a biocompatible method to protect bacteriophages from the adverse effects of UV exposure. The hypothesis is that such protection is necessary for the simultaneous actions of both phages and UV against bacteria. Without such protection, UV deactivates phages before they can infect host cells. UV offers robust disinfection but is limited in effectiveness within opaque media and confined spaces. In contrast, while requiring longer incubation times, phages are highly selective and can act in areas inaccessible to light. Our results distinguished brilliant blue FCF (BB) as a selective stabilizer that protects bacteriophages from the adverse effects of UV irradiation while remaining neutral to gram-negative bacteria. BB is harmless to mammalian cells [[32], [33], [34]].

BB-stabilized bacteriophages presented significantly higher efficacy in UV-related protocols and environmental applications than non-protected virions. We demonstrated membrane and food sterilization by the dual action of phages and UV light. These applications were chosen as foodborne pathogens, such as *Escherichia coli* and *Salmonella enterica*, which are among the leading causes of foodborne illnesses worldwide, contributing significantly to public health concerns and economic losses. Biofilms formed by these and other bacteria exacerbate contamination risks by protecting conventional cleaning and disinfection methods. Addressing biofilm-associated contamination is, therefore, critical for enhancing food safety and reducing the burden of foodborne diseases.

## 2 MATERIALS AND METHODS

### 2.1 Chemicals and biological media

The protective properties against UV radiation were examined using eight popular, commercially available food dyes – brilliant blue FCF (BB, E133), azorubine (AZ, E122), tartrazine (TR, E102), quinoline yellow (QY, E104), sunset yellow FCF (SY, E110), Allura red (AR, E129), Ponceau 4R (PC, E124), and indigocarmine (IC, E132). All the dyes, except for the Allura red, were provided by Food Colours Perczak S.J. (Piotrków Trybunalski, Poland). Allura red dye was provided by Sweetdecor S.C. (Radzików, Poland). Unless stated otherwise in the text, all the dyes were used at 0.5 % (w/v) concentration.

Sodium dodecyl sulfate (SDS; Sigma-Aldrich, Saint Louis, Missouri, USA), 2-(*N*-morpholino)ethanesulfonic acid (MES, Sigma-Aldrich, Saint Louis, Missouri, USA), toluene sulfonic acid (TSA; POCH, Gliwice, Poland), 4-hydroxybenzenesulfonic acid (HBSA; TCI Europe N.V., Zwijndrecht, Belgium), sodium 2-naphthol-6-sulfonate (NSA; TCI Europe N.V., Zwijndrecht, Belgium), and sodium isatin-5-sulfonate (ISA; Sigma-Aldrich, Saint Louis, Missouri, USA), isatin (Sigma-Aldrich, Saint Louis, Missouri, USA) were used as received. Before the experiment, the acidic compounds (toluene sulfonic acid and 4-hydroxybenzene sulfonic acid) were neutralized using 0.1 M NaOH solution.

LB-agar contained 15 g/L of agar, 10 g/L of NaCl, 10 g/L of tryptone, 5 g/L of yeast extract, and 15 g/L of agar (Carl Roth, Germany), and it was used as an instant mix (Carl Roth, Germany). LB Top-Agar had the same composition, except that the agar concentration was 5 g/L. Liquid LB-medium had the same composition except for lacking 15 g/L of agar (Carl Roth, Germany).

TM buffer (pH = 7.4) was formulated using 10 mM Tris base, 5 μM CaCl_2_, 10 mM MgSO_4_, and distilled water, with its components obtained from Sigma Aldrich. All solutions underwent sterilization through autoclaving before use. A phosphate buffer (50 mM, pH 7.4) was prepared from NaH_2_PO_4_ and Na_2_HPO_4_, sourced from Carl Roth (Karlsruhe, Germany).

### 2.2 Instrumentation

CL-1000 Ultraviolet Crosslinker, UVP; 5 × 8 W, 254 nm UV, was used. In most experiments, phages and bacteria were exposed to UV for 1 min, and the energy was around 360 mJ/cm^2^.

Phage suspensions were exposed to artificial sunlight using Wavelabs LS-2 AAA, 160 × 160 mm^2^ LED solar simulator (Wavelabs, Hyderabad, India), intensity = 100 %.

### 2.3 Bacteriophages and bacteria used in the study

Representatives of non-enveloped bacteriophages – T4 (*Straboviridae*), T7 (*Autographiviridae*), MS2 (*Leviviridae*), M13 (*Inoviridae*) (obtained from the collection from the Institute of Physical Chemistry of Polish Academy of Sciences, Warsaw, Poland), LR1_PAO1 (*Myoviridae*) (isolated from the Baltic sea water), vB_SauS_CS1 (*Siphovirus*), P22 (*Podoviridae*) (obtained from the German Collection of Microorganisms and Cell Cultures, Braunschweig, Germany), and Phi29 (*Salasmaviridae*) (obtained from the German Collection of Microorganisms and Cell Cultures, Braunschweig, Germany) were tested. As the host for selected bacteriophages, *E*. *coli* BL21 (obtained from the collection of the Institute of Biochemistry and Biophysics, Warsaw, Poland; T4 and T7 bacteriophage), *E*. *coli* C3000 (obtained from the collection of the Institute of Biochemistry and Biophysics, Warsaw, Poland; MS2 and M13 bacteriophages), *Pseudomonas aeruginosa* PAO1 (obtained from the collection of the Institute of Biochemistry and Biophysics, Warsaw, Poland; LR1_PAO1 phage), *Staphylococcus aureus* DSM 105272 (obtained from the German Collection of Microorganisms and Cell Cultures, Braunschweig, Germany; vB_SauS_CS1 bacteriophage), *S*. *enterica* DSM 18522 (obtained from the German Collection of Microorganisms and Cell Cultures, Braunschweig, Germany; P22 bacteriophage), and *Bacillus subtilis* DSM 5547 (obtained from the German Collection of Microorganisms and Cell Cultures, Braunschweig, Germany; Phi29 bacteriophage) were used.

In the antibacterial assays, *E. coli* BL21 (obtained from the Institute of Biochemistry and Biophysics in Warsaw, Poland) and *Acinetobacter baumannii* ATCC BAA-1605 (obtained from Pomeranian Medical University, Szczecin, Poland) were used as representatives of gram-negative bacteria. *Bacillus subtilis* DSM 5547 (obtained from the German Collection of Microorganisms and Cell Cultures, Braunschweig, Germany) and *S. aureus* ATCC 43300 (obtained from Pomeranian Medical University, Szczecin, Poland) strains were used as gram-positive bacteria representatives.

### 2.4 Bacteriophage protection against UV radiation

The experiments were performed according to the previously described protocol [22] with minor modifications. In short, bacteriophages were diluted in the TM buffer solution (TRIS, magnesium sulfate, 10 mM) to reach the initial titer of 10^5^ PFU/mL (plaque-forming units per milliliter). Then, 5 mg of selected food dye was added to 1 mL of phage suspension to reach a concentration of around 0.5 % (w/v), and the mixtures were incubated at 4 °C for 24 h. After the incubation, the quantitative analysis of bacteriophage activity was performed using the droplet test on double-layer agar. The top agar layer contained host cells of *E*. *coli* BL21, *E. coli* C3000, or *S*. *enterica* DSM 18522. A droplet test was performed by placing at least eight droplets (5 μL each) of each phage suspension on the top agar layer. The remaining bulk of each sample was transferred to sterile glass Petri dishes (45 mm diameter). Irradiation was performed by placing glass Petri dishes inside a UV chamber for 1 min, with plates positioned 15 cm from the radiation source. The second titration occurred after all the samples were exposed to UV-C radiation (λ = 254 nm) for 1 min.

### 2.5 BB-stabilized phages exposed to sunlight

To verify if the BB-stabilized bacteriophages would be effective in natural environment conditions, T4 bacteriophages were suspended in the TM buffer solution, 0.5 % of BB in the TM buffer solution, to reach a concentration of about 10^6^ PFU/mL. After the overnight incubation, 1.98 mL of each suspension was transferred to the quartz cuvettes (10 × 10 mm, Hellma). Phage suspensions were exposed to artificial sunlight. 300 μL of each solution was transferred to a fresh Eppendorf tube at the beginning of the experiment (0′), after 30 min of exposure (30′), and at the end of the experiment (60’).

### 2.6 Bacteria protection against UV radiation

The bacteria were cultured according to the plating method protocol. The single colony from the culture on the LB agar plate was inoculated into 10 mL of LB and then incubated at 37 °C in an orbital shaker (220 RPM) overnight (about 16 h). Next, the overnight cultures were refreshed by adding 7.5 mL of fresh LB medium to 2.5 mL of the overnight culture and incubated for 1 h at 37 °C. Then, the refreshed cultures were both diluted with LB to reach an optical density of around OD_600_ = 1.0 for *E. coli* (about 8.0 × 10^8^ CFU/mL) (colony-forming units per milliliter), OD_600_ = 1.0 for *A*. *baumannii* (about 5 × 10^9^ CFU/mL), OD_600_ = 1.0 for *B*. *subtilis* (about 1.0 × 10^8^ CFU/mL), or OD_600_ = 1.0 for *S*. *aureus* (about 5.0 × 10^9^ CFU/mL). Afterward, the bacteria were diluted in a phosphate buffer with or without 0.5 % (w/v) of adequate food dye to reach the initial concentration of 10^5^ CFU/mL. Before 24 h of incubation at room temperature with shaking (220 RPM), 100 μL of each solution was cultured onto LB agar plates. After incubation, another 100 μL of bacterial suspension was cultured on fresh LB agar plates to investigate if the food dyes affected bacterial growth. The remaining samples were transferred to sterile glass Petri dishes (45 mm diameter) and exposed to UV-C radiation for 1 min inside the UV chamber. After the exposure, 100 μL of the bacterial suspension was cultured on fresh LB agar plates. In each case, the plate count method was used to determine the viability of the bacteria.

### 2.7 Sterilization of syringe filter membranes

The experiments were performed according to the protocol described previously [22]. In short, Nylon66 syringe filters were used as a simplified model for sterilizing the membrane surfaces. First, refreshed *E. coli* BL21 (OD_600_ = 0.9) or *B. subtilis* DSM 5547 (OD_600_ = 1.27) suspensions were diluted in the TM buffer to obtain a concentration of bacteria of about 10^5^ CFU/mL. The Nylon66 filters were flushed with 5 mL of such bacterial cultures. The same volume was flushed through the filter twice. Then, the filters were filled with 1 mL of the following samples and exposed to the following conditions: 1) control (TM buffer); 2) TM buffer and 10 min of UV exposure; 3) TM buffer and T4/Phi29 phages; 4) BB; 5) TM buffer, T4/Phi29, and UV; 6) BB, and UV; 7) T4/Phi29 and BB; 8) T4/Phi29, BB, and UV. The final concentrations were around 10^4^ PFU/mL of T4/Phi29 phages, 0.1 % BB. The samples were incubated for 20 min at room temperature and then exposed for 10 min to UV radiation within the laminar hood (Thermo Scientific MSC-ADVANTAGE; 36 W, 254 nm UV). The filters were washed with 1 mL of TM buffer solution to recover bacterial cells. The number of bacteria was estimated using the plating method. After the overnight incubation at 37 °C, colonies were counted. For both control samples, a concentration of about 5 × 10^4^ CFU (*i.e.*, 5 mL of 10^4^ CFU/mL suspension) was introduced, while approximately 4.8 × 10^4^ CFU was recovered.

### 2.8 BB-stabilized phages in food preservation

To prove the efficacy of BB-stabilized phages in food preservation, we performed experiments simulating bacterial contamination on the surface of lettuce leaves. *E. coli* BL21 and *S. enterica* DSM 18522 (both gram-negative) were used because these bacterial species are the most frequent contaminants of plant-based food. Lettuce leaves were cut with a sterile scalpel into 1.5 cm × 1.5 cm square pieces, and then 100 μL of 10^5^ CFU/mL bacterial suspension (*E. coli* or *S. enterica*) was applied on the surface of the lettuce pieces. The pieces of leaves were left at room temperature until the suspension dried. Then, 100 μL of T4/P22 (against *E. coli* and *S. enterica*, respectively) phage suspension (about 10^6^ PFU/mL) in TM buffer solution or TM buffer with 0.5 % BB was applied onto the surface of the lettuce pieces. As a control, 100 μL of TM buffer solution was used. The samples were incubated for 25 min at room temperature, then exposed for 5 min to UV radiation within the laminar hood (Thermo Scientific MSC-ADVANTAGE; 36 W, 254 nm UV), and homogenized with the kitchen mortar. Homogenized samples were suspended in 1 mL of fresh TM buffer solution, from which 100 μL were cultured and incubated overnight on LB-agar plates at 37 °C. After the incubation, colonies were counted.

### 2.9 Double cuvettes experiment

1 mL of T4 bacteriophage suspensions, first incubated overnight at 4 °C in TM buffer, was placed in quartz cuvettes (to provide the transparency of the glassware to UV and eliminate the risk of free radicals generation from plastic). Then another set of quartz cuvettes was placed on top of the cuvettes containing phage suspension. In one sample, the cuvette was empty, another one was filled with 300 μL of TM buffer solution, and the third was filled with 300 μL of 0.5 % (w/v) brilliant blue FCF solution. The quartz cuvettes were joined using parafilm. Then, they were covered with aluminum foil so the light reached the phage suspension only from above. Then, the experiment was performed according to the protocol described in the ‘**Bacteriophage protection against UV radiation’** section.

### 2.10 Statistical analysis

All experiments were performed in triplicates. Statistical analysis was performed using the Students’ t-test to determine if the observed differences were statistically significant (∗p < 0.05; ∗∗p < 0.01; ∗∗∗p < 0.001).

## 3 Results and discussion

### 3.1 Bacteriophage and bacteria protection against UV irradiation

**Fig. 1:**
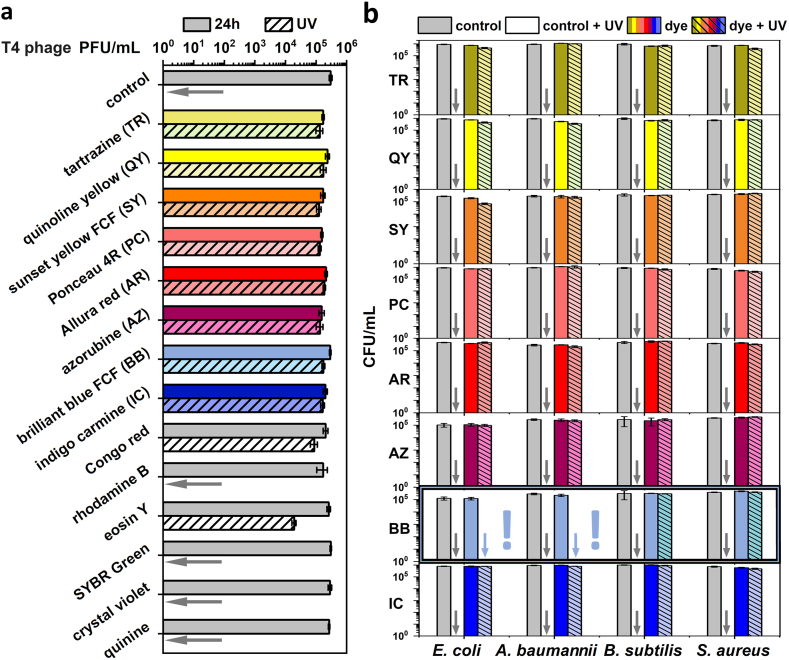
**a)** The evaluation of UV-protective properties of the food dyes to T4 phages. The overnight incubation in 0.5 % solutions of tartrazine (TR), quinoline yellow (QY), sunset yellow FCF (SY), Ponceau 4R (PC), Allura red (AR), azorubine (AZ), brilliant blue FCF (BB) and indigo carmine (IC) prevented the titer drop caused by 1 min UV exposure. Additional control compounds were tested, i.e., Congo red, rhodamine B, eosin Y, SYBR Green, crystal violet, and quinine. **b)** TR, QY, SY, PC, AR, AZ, and IC protected both gram-negative (*E. coli, A. baumannii*) and gram-positive (*B. subtilis, S. aureus*) bacteria. Only BB was selective, i.e., inefficient in protecting gram-negative bacteria. (For interpretation of the references to color in this figure legend, the reader is referred to the Web version of this article.)

We exposed T4 phages to UV dose, causing complete (>5 log, i.e., below the methods’ detection limit <25 PFU/mL) deactivation of virions (1 min, ∼360 mJ/m^2^). All eight judiciously chosen food dyes (tartrazine (TR), quinoline yellow (QY), sunset yellow FCF (SY), Ponceau 4R (PC), Allura red (AR), azorubine (AZ), brilliant blue FCF (BB) and indigo carmine (IC) provided nearly perfect protection to bacteriophages (Fig. 1a). To verify if virus protection is a common trait of dyes, we also tested Congo red, rhodamine B, eosin Y, SYBR Green, crystal violet, and quinine to cover a wide variety of different structures and properties. Among the tested dyes, Congo red provided adequate protection to phages, which aligns with our previous reports [22]. Previously, it was found that CR specifically binds to proteins in the beta-sheets-rich fragments of the proteins [35] and that this property is crucial for bacteriophage stabilization against UV irradiation [22]. Eosin Y provided some protection against the UV, but a decrease in phage titer larger than 1-log was observed. Crystal violet binds to the bacterial cell walls (e.g., in Gram staining), which are absent in bacteriophage virions. The lack of efficacy of SYBR and quinine strongly suggests that binding to the DNA is not related to the stabilization against irradiation. Rhodamine B is a commonly used fluorescence dye known to interact with membranes [36] and genetic material [37], and thus, it also did not show any phage-stabilizing effect (Fig. 1a). All molecular structures of the studied compounds are given in the **Supporting** **Information**.

Congo red was deemed hazardous and potentially harmful to the environment and animals [23], but was also a less efficient phage protectant than the chosen dyes. For all selected food dyes, the mean EC_50_ values, i.e., the concentration that protects 50 % of virions (Figures S1-S8), were significantly lower (around 0.1 % – 0.3 %) compared to Congo red (0.7 % – 1.1 %, depending on the virus). A summary of the efficacy of all tested food colorants in protecting virions against UV irradiation is given in the **Supporting** **Information**.

The tests on bacteria revealed that most food dyes also protected bacteria. All tested food dyes provided protection against UV to gram-positive bacteria. Only BB did not protect gram-negative bacteria (Fig. 1b). This proved that selectivity in such systems is rare, and the mechanism behind the protection requires deeper understanding. Our previous findings showed that certain gram-negative bacteria, such as *Pseudomonas aeruginosa*, possess intrinsic UV resistance due to pigment production (e.g., pyoverdine, pyocyanin). Still, this natural protection did not influence dye activity, as Congo red failed to enhance UV resistance of these bacteria [22].

**Fig. 2:**
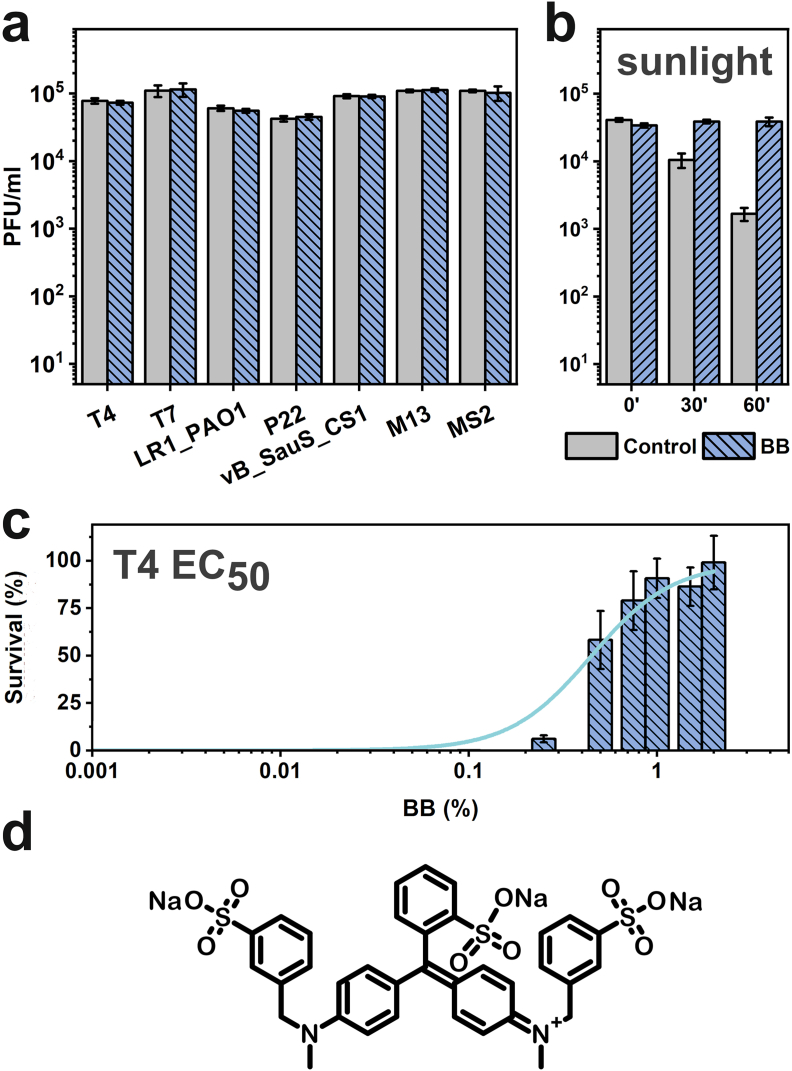
**a)** BB was effective in protecting a plethora of bacteriophages, including T4, T7, MS2, M13 (host: *E. coli*), P22 (host: *S. enterica*), vB_SauS_CS1 (host: *S. aureus*) and LR1_PAO1 (phage isolated from the environment, host: *P. aeruginosa*). BB negatively impacted enveloped phage Phi6 (host: *P. syringae*) (data not shown). **b)** BB prevented the titer drop upon exposure of T4 phages to artificial sunlight. **c)** EC_50_ concentration of BB was established (T4 - 0.32 %, MS2 - 0.42 %, M13–0.41 %, P22–0.32 %). Here, exemplary data for T4 was shown. **d)** Molecular structure of BB.

For all further studies, we focused on BB (Fig. 2d) as a formulation component, selectively protecting phages against UV. First, we tested the protective properties of BB on other phages. All non-enveloped phages were protected against UV (Fig. 2a). LR1_PAO1 is a phage isolated from the environment with *P*. *aeruginosa* as a host. vB_SauS_CS1 is *S*. *aureus* phage. P22 infects *S*. *enterica*. Values of EC_50_ of BB varied slightly depending on the phage (T4 - 0.32 %, MS2 - 0.42 %, M13–0.41 %, P22–0.32 %). The exemplary dependence of survival rate and BB concentration is given in Fig. 2c. Even without UV, BB inactivated Phi6 – an enveloped phage (not shown).

We also exposed T4 phages to sunlight at room temperature to mimic the potential application in agriculture. It appeared that sunlight had a negative effect on the titer. The addition of BB stabilized phage suspension, and no decrease in titer was observed (Fig. 2b).

**Fig. 3:**
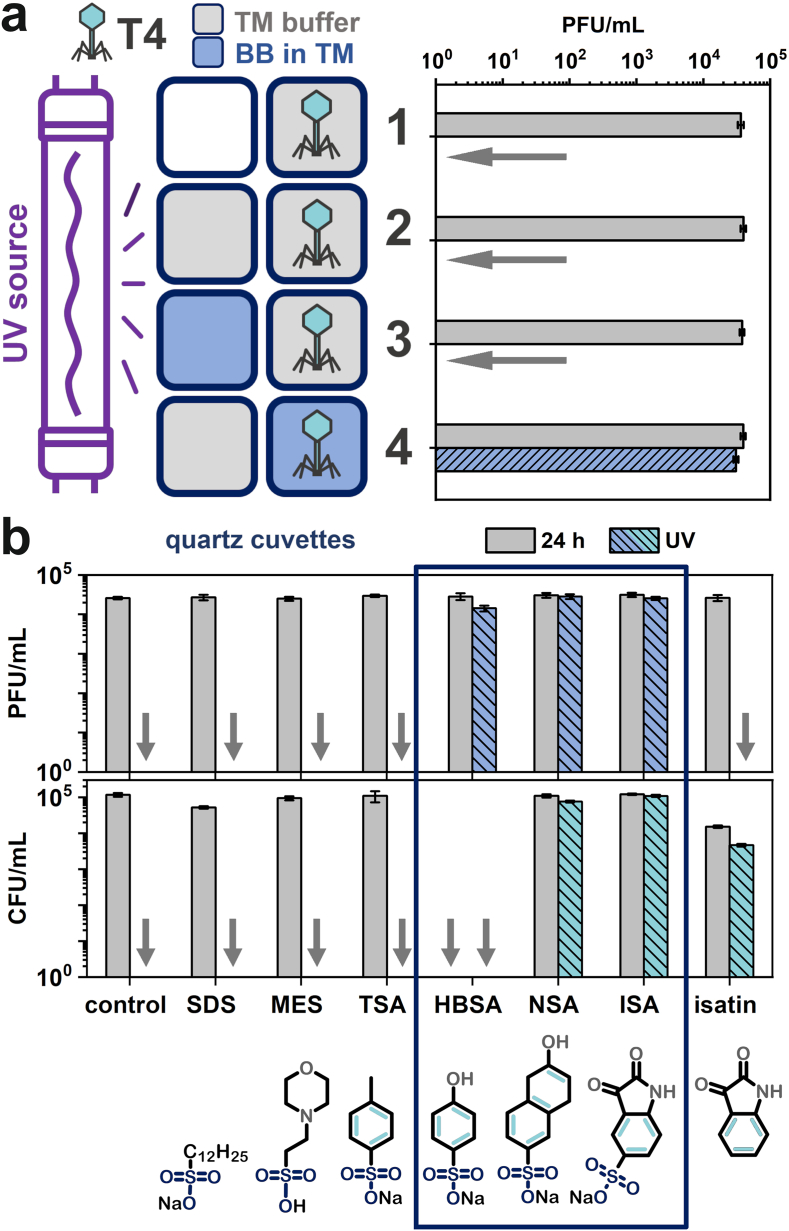
**a)** The evaluation of the molecular basis of dye-mediated UV protection of bacteriophages. The schematic illustration of experiments in which phages were either mixed with BB or placed in separate cuvettes. Phages were protected only when BB was added directly to the suspension. **b)** The evaluation of phage (T4)/bacteria (*E. coli*) -protective properties of sodium dodecyl sulfate (SDS), 2-(*N*-morpholino)ethanesulfonic acid (MES), sodium toluene sulfonate (TSA), sodium 4-phenol sulfonate (HBSA), sodium 2-naphthol-6-sulfonate (NSA), sodium isatin-5-sulfonate (ISA), and isatin along with the molecular structures of the compounds. The analysis allowed us to identify three domains required for the molecule to have phage-protective properties: *i)* sulfonic group, *ii)* UV-absorbing domain (here – aromatic rings), and *iii)* side group able to form hydrogen bonds.

We aimed to unravel the protective mechanism of BB action and took a step forward to understand the selectivity in phage/bacteria systems. We designed a simple yet elegant experiment to prove that interactions between BB and virions are substantial for protection (Fig. 3a). Two quartz cuvettes were bonded and covered so light could only pass through from one direction. Phages remained active only when BB was added directly to the suspension. No protection was observed when BB was placed in the first cuvette and phages in the second one. This confirmed that the mechanism was not simply the absorption of UV light by a solution of dye. If this were true, all UV-absorbing molecules should protect virions, which was not the case (Fig. 1a). The necessity of placing both components in a single cuvette was a strong suggestion that the UV protective properties required interactions between virions and protectants. We postulate that the protection originated in food dyes binding to the surface of the viral capsid, which resulted in the accumulation of molecules, absorption or scattering of a significant portion of photons, and finally, reduction of the exerted impact of UV irradiation on viral particles. We propose the name ‘molecular sunscreen’ mechanism.

It was unclear why some molecules can protect virions against UV while others do not. We analyzed the molecular structures of compounds studied in Fig. 1a (i.e., both selected food dyes and reference molecules). All compounds providing adequate protection to virions appeared to have at least one sulfonic group. Eosin Y did not contain a sulfonic group but offered much weaker protection than food dyes. To verify that this was also true in the case of bacteria, we compared the protective properties of BB and crystal violet (CV). CV stains bacterial cell walls in a protocol known as Gram staining [38], but it does not possess any sulfonic group. CV presented no stabilizing properties towards the examined bacteriophage (T4, Fig. 1a) and bacteria (*E. coli, S. aureus*; Fig. S9). We went further and compared the behavior of sodium isatin-5-sulfonate (ISA) and isatin. These two compounds differ only by a single sulfonic group. ISA (with sulfonic group) protected T4 virions, while isatin (without sulfonic group) did not (Fig. 3b).

Other reports hinted previously at the importance of the sulfonic groups in phage-interacting systems (both colloidal and molecular) [39,40]. However, just the presence of sulfonic groups was clearly not enough to explain the observed phenomena - sodium dodecyl sulfate (SDS), 2-(*N*-morpholino)ethanesulfonic acid (MES), and sodium toluene sulfonate (TSA) did not provide any protection against UV to neither phages nor bacteria (Fig. 3b). Only more complex sodium 4-phenol sulfonate (sodium hydroxybenzenesulfonate; HBSA), sodium 2-naphthol-6-sulfonate (NSA), and sodium isatin-5-sulfonate (ISA) offered beneficial properties for phage stabilization.

The results suggested that there are three domains in the molecular structure for a compound to protect phages against UV: *i)* –SO_3_^-^ group; *ii)* the aromatic domain absorbing the photons and dissipating the energy [41]; *iii)* additional hydrophilic group (such as –OH) to form hydrogen bonds.

SDS and MES showed very low absorbance both in the UV and visible range and thus did not affect phage stability upon UV irradiation (Fig. S10) It should be noted that we did not find any apparent relationship between extinction coefficient and magnitude of protection as long as the absorption is non-zero (also in the case of food dyes). Only a slight change in the structure, i.e., replacing the –CH_3_ group (TSA) with the –OH group (HBSA), resulted in completely different results. We assumed that the possibility of hydrogen bond formation was also a crucial requirement for the compound to protect phages. This was confirmed as not only HBSA but also NSA and ISA were active in our experiment, maintaining virion stability upon UV exposure. Additionally, when chaotropic SDS (0.1 %) was added to BB (at EC_50_), the number of UV-surviving phages dropped by around 1-log compared to only BB. This again proved the importance of hydrogen bonds for such systems.

It is known that sulfonic groups bind to proteins' protonated amino acid side chains (NH_3_^+^ in lysine, arginine, and histidine) [29] with additional stabilizing hydrogen bond formation with tyrosine [42]. We found out that basic amino acids constitute about 10 % of proteins present at the surface of studied viruses and gram-positive bacteria (T4 major capsid protein gp23, *Salmonella* P22 phage major capsid protein, *Escherichia* phage MS2 capsid protein, Enterobacterial phage M13 capsid protein G8P, major surface proteins of *S. aureus*, ligand-binding A-domain of SasG protein). However, in the case of *E. coli* (OmpA extracellular domain), the value is significantly lower, i.e., 5.9 %. Therefore, there were more binding sites on the surface of virions and gram-positive bacteria than gram-negative bacteria. We hypothesized that this may be one of the factors determining the BB selectivity shown in the study. The fact that sulfonic compounds (with an emphasis on 2-naphthol-6-sulfonate and isatin-5-sulfonate) by default weren't selective (Fig. S11), similar to the remaining food colorants, indicated the need for a better understanding of interactions between biomolecules and sulfonates to facilitate biotechnological applications. It should be noted that the lack of activity of CV (Fig. 1a and Figure S9) showed that only binding to the surface was not enough to provide protection against UV.

It is important to note that attempts to remove unbound BB from phage preparations, whether *via* dialysis, ultracentrifugation, or reverse osmosis, would be both technically challenging and economically prohibitive and could inadvertently reduce the bound fraction essential for UV protection. Because the protective effect of BB relied on its direct association with the phage virion, removing unbound dye would likely reduce the number of bound molecules and compromise UV protection. Dilution in the fresh buffer would shift the binding equilibrium, leading to progressive BB detachment over time and reduced efficacy. Importantly, BB is a food-grade dye approved for use in consumables, and its residual presence in formulations is acceptable for food preservation and related applications. Therefore, maintaining BB in the solution offers functional stability and practical feasibility for real-world deployment.

### 3.2 The sterilization of membranes

**Fig. 4:**
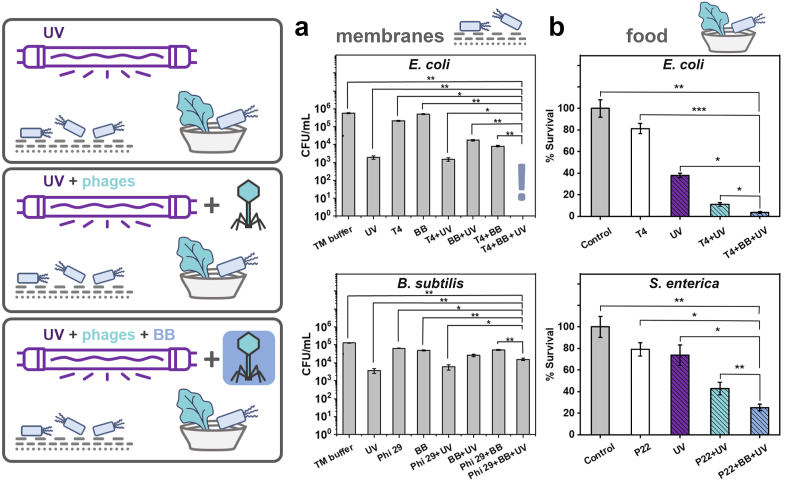
**a)** The application of BB-stabilized bacteriophages for the dual-mode (phages + UV) sterilization of membranes. The surfaces of syringe filter membranes were spiked with bacteria (*E. coli* or *B. subtilis*), and then BB-stabilized bacteriophages (T4 or Phi29, respectively) were introduced to the filters. UV irradiation was used as in previous experiments. **b)** The simultaneous application of UV and phages stabilized with BB for food sterilization against bacterial contamination. The sterilization of lettuce leaves surface spiked with *E. coli* or *S. enterica* (both gram-negative) contamination showed significantly better results when BB was added to T4 or P22 phages, respectively. More detailed statistical analysis is presented in Tables S1–S4 (∗p < 0.05; ∗∗p < 0.01; ∗∗∗p < 0.001).

Membranes contaminated with bacteria were exposed to UV irradiation and BB-stabilized bacteriophages. We found that only protected phages in combination with UV allowed for the complete eradication of gram-negative bacteria. The results are presented in Fig. 4a.

The sterilization protocol consisted of a 20-min incubation in bacteriophage (10^4^ PFU/mL) suspensions followed by 10 min of exposure to UV irradiation. The experiment revealed significant differences in results depending on the bacterial contaminant species. For the *E. coli* (gram-negative), the BB itself had a neutral effect, as expected. Only UV irradiation resulted in a 3-log reduction in bacterial titer, while T4 phages alone had a relatively small impact on the number of bacteria. However, combining UV irradiation and BB-stabilized T4 phages reduced the bacteria titer to below the method's detection limits (about 10 CFU/mL). The effect was synergistic and not simply additive.

For *B. subtilis* (gram-positive), the UV irradiation resulted in a 2-log reduction in bacterial titer. The combination of UV and Phi29 bacteriophage (specific for the tested bacteria) did not improve the effect. When the combination of UV irradiation and BB-stabilized Phi29 bacteriophages was applied, the decrease of phage titer was about 1-log, so the performance was worse compared to the effect of only UV irradiation.

### 3.3 BB-stabilized phages in food preservation

We examined the efficacy of bacteriophages stabilized with BB for food preservation. *E. coli* and *S. enterica* (both gram-negative), i.e., the most frequent bacterial food contaminants. 1.5 cm × 1.5 cm pieces of lettuce leaves were spiked with 100 μL of 10^5^ CFU/mL bacterial suspension. Next, UV irradiation, adequate bacteriophages (T4 or P22), and their combinations were applied. The results are presented in Fig. 4b. The mix of UV irradiation and bacteriophages was more effective in bacteria elimination (about 85 % reduction for *E. coli* and 60 % reduction for *S. enterica*) than the UV and bacteriophages separately. However, thanks to the addition of BB-stabilized phages, the efficacy of such a protocol was significantly better (p < 0.05 or p < 0.01) in the case of both *E. coli*, with approximately 94 % reduction of the bacterial count, and *S. enterica*, with approximately 75 % reduction of the bacterial count.

BB is generally considered a non-toxic dye and is already used for staining numerous brands of candies, soft beverages (including popular brands such as Gatorade), alcoholic drinks (Blue curaçao liqueur), and cosmetic requisites such as mouthwash or after-shave products [43]. Studies on animal models showed that BB did not present fetal toxicity when applied orally [32], and its absorption in the intestines was about 0.27 %. Most of the up-taken dye was removed from the organism with urine [44]. In our tests, BB also appeared biocompatible and non-hazardous to human cells (cf. **Supporting** **Information**).

Furthermore, there is almost no risk that BB would affect the efficacy of photosynthesis and plant metabolism. The dye's maximum absorbance (app. 620 nm) didn't overlap with the absorption spectra of chlorophyll *a* (420 nm, 680 nm) and chlorophyll *b* (480 nm, 640 nm) [45]. The harmlessness of Brilliant blue-group dyes towards plants has already been proved [33,46].

In the bacteria eradication experiments (i.e., membrane and food sterilization), bacteria were not separated from phages before CFU enumeration. However, all samples, including controls without UV exposure, underwent identical treatment and plating protocols, which accounts for the potential effects of residual phage activity during incubation. Moreover, separating bacteria from phages is technically challenging. Adsorbed phages would be pelleted together with bacterial cells during centrifugation, and infected cells carrying unreleased phage progeny would not be distinguishable from uninfected bacteria. Therefore, our approach reflects a realistic and controlled scenario for evaluating treatment efficacy.

## 4 Conclusions

Efficient stabilizing protocols are essential to fully embrace bacteriophages' antibacterial potential. This is particularly important when phages are *i)* exposed to sunlight (e.g., in agriculture) or *ii*) are to be used simultaneously with UV for sterilization. We provided two examples of the simultaneous action of phages and UV for membrane and food sterilization. We envision that the developed method might also be used in agriculture. Plants (especially fruit plants and vegetables) are also threatened by bacterial infection. Bacterial plant diseases are estimated to cause annual economic losses of about 220 billion USD globally [47]. Among numerous bacterial pathogens, *E. amylovora* (the etiological factor of fire blight disease of apple trees) [48], *Agrobacterium tumefaciens* [49], *X. fastidiosa* [50], and *P. syringae* [51] are responsible for the most important, from the economic point of view, diseases. All these bacteria are gram-negative.

Although the experiments in this study were conducted under laboratory conditions, the tested scenarios, such as food surface decontamination and membrane sterilization, closely mimic realistic applications where phages and BB are applied together in a controlled volume. BB has been approved for industrial use as a food-grade dye, supporting its regulatory compatibility.

We found that a dose of UV radiation frequently used for sterilization was sufficient to inactivate virtually all bacteriophages (5-6-log reduction of phage titer) within 1 min. Adding 0.5 % of examined food dyes reduced the decrease to below 0.5-log (Fig. 1a). We estimated that the EC_50_ varied from 0.11 % (tartrazine and T4 phages) to 0.42 % (brilliant blue and MS2 phages) (Figs. S1–S8), i.e., significantly lower compared to the EC_50_ previously reported for Congo red (ranging from 0.7 % to 1.11 %, depending on the bacteriophage [22]). Compared to the previous study on enveloped viruses [40], no food dyes appeared as the *antiphagent*, indicating no antiviral activity towards non-enveloped viruses.

Several key criteria drove our selection of dyes. First, we focused on food-grade dyes, as their approval for use in multiple applications ensures biocompatibility and safety. The food dyes selected for this study are internationally approved for use by proper food-related institutions, including the Food and Drug Administration (FDA) and the European Food Safety Authority (EFSA). Second, based on previous studies, we hypothesized that the presence of sulfonic groups is critical because these groups bind to proteins' protonated amino acid side chains and form additional stabilizing hydrogen bonds with tyrosine [29,42], a notion supported by other reports on phage-interacting systems [39,40]. Third, we intentionally tested molecules with known affinity for specific domains: for example, Congo red was selected for its specific binding to beta-sheet-rich protein fragments, an interaction pivotal for phage stabilization [22,35], while crystal violet, SYBR green, and rhodamine B, known respectively for binding to bacterial cell walls, DNA, and membranes/genetic material [36,37], were included as controls. Finally, we selected dyes that are highly soluble in water to ensure compatibility with aqueous formulations. In Fig. 1, the tested molecules include tartrazine, quinoline yellow, sunset yellow FCF, Ponceau 4R, Allura red, azorubine, brilliant blue FCF, and indigo carmine; additionally, we included Congo red, rhodamine B, eosin Y, SYBR green, crystal violet, and quinine as control compounds. This diverse panel enabled us to investigate the structural features that govern the ‘molecular sunscreen’ effect and its impact on phage stabilization under UV irradiation.

Only brilliant blue FCF (BB) was selective and did not provide any protection to the representatives of gram-negative bacteria (*E. coli* and *A. baumannii*) (Fig. 1b). BB is generally considered non-toxic and broadly used in the food industry. Our protocol was equally effective in laboratory and environmental conditions, as it was under sunlight exposure (Fig. 2b). These results suggested that adding this food dye could be crucial to effectively deal with gram-negative bacterial contaminations, which are the most common contaminants in agriculture, biofilm formation, and food spoilage [[52], [53], [54]].

Knowing the optimal concentrations of BB dye and the mechanism behind its protective properties (i.e., ‘molecular sunscreen’), we verified the potential of BB-stabilized virions in membrane sterilization and food preservation. We confirmed that without BB, there was almost no benefit to adding phages to UV treatment. This was because UV deactivated virions before they could infect bacteria and cause cell lysis. Combining all three components, i.e., phages, UV, and protectant, allowed for eradicating gram-negative bacteria in the membrane systems and sterilizing food (Fig. 4).

The structural diversity of bacteriophages raises the question of how broadly applicable BB-based protection may be. Tailed phages, such as T4, represent over 95 % of known bacteriophages [55], and their complex surface architecture typically includes positively charged domains, particularly at the tail fibers, which facilitate electrostatic attraction to negatively charged bacterial surfaces. Our analysis of T4, a well-characterized tailed phage, along with successful BB-mediated protection of filamentous (M13) and spherical (MS2) phages, indicates that BB's protective effect is not restricted to a specific capsid morphology. Given that efficient host binding requires some degree of surface positivity, it is likely that many phages, regardless of morphology, expose regions enriched in basic residues, supporting the broader applicability of this protective strategy.

This study is exploratory in nature and provides a generalizable formulation concept rather than a fixed-dose protocol. Specific BB concentrations should be optimized for each application. Notably, in the use cases we investigated, such as food surface decontamination and membrane sterilization, BB and phages are applied together in a defined liquid volume, eliminating variability in local concentration. For formulations intended for broader or systemic use, such as in suspensions or packaged products, further adjustment of BB levels may be required to maintain the selectivity of the protective effect.

To conclude, this study showed that food colorants can successfully protect non-enveloped (i.e., the vast majority [55]) bacteriophages against UV inactivation. This protection was specific only for brilliant blue FCF and allowed the simultaneous use of UV and phages to eliminate contaminations caused by gram-negative bacteria (Fig. 4). Hence, *E. coli and B. subtilis* are the most commonly used model species of bacteria. Our conclusions can generally pertain to other bacteria, including plant pathogens. We strongly believe that all these advancements are crucial for facilitating the application of bacteriophages as antimicrobials in the post-antibiotic era that we might face soon.

## CRediT authorship contribution statement

**Mateusz Wdowiak:** Writing – review & editing, Writing – original draft, Visualization, Investigation, Data curation, Conceptualization. **Aneta Magiera:** Writing – review & editing, Writing – original draft, Investigation, Data curation. **Magdalena Tomczyńska:** Investigation, Data curation. **Witold Adamkiewicz:** Writing – review & editing, Writing – original draft. **Francesco Stellacci:** Writing – review & editing, Writing – original draft, Validation, Formal analysis. **Jan Paczesny:** Writing – review & editing, Writing – original draft, Visualization, Validation, Supervision, Project administration, Methodology, Funding acquisition, Formal analysis, Conceptualization.

## Funding sources

The research was financed by the 10.13039/501100004442National Science Centre, Poland, within the OPUS grant number 2022/45/B/ST5/01500. A.M. was supported by the 10.13039/501100001870Foundation for Polish Science within the START scholarship.

## Declaration of competing interest

The authors declare that they have no known competing financial interests or personal relationships that could have appeared to influence the work reported in this paper.

## Data Availability

All the data and documentation are stored in the online Repository for Open Data (RepOD); https://doi.org/10.18150/AAA8KM.

## Appendix A Supplementary data

Supporting **Information** contains the list of chemicals, microorganisms, and viruses used in the study, an estimation of EC_50_ values for all examined dyes, a detailed explanation of the mechanism behind the dye-mediated UV protection, and the cytotoxicity assay verifying the biocompatibility of our BB dye.
