# Supplementary material for: Protecting bacteriophages under UV irradiation with brilliant blue FCF for targeted bacterial control
Source: Biofilm. 2025 May 9;9:100286. doi: 10.1016/j.bioflm.2025.100286 (PMC12143832; doi:10.1016/j.bioflm.2025.100286)
Supplement: Multimedia component 1 [file mmc1.docx]

Supporting Information

Protecting Bacteriophages Under UV Irradiation with Brilliant Blue FCF for Targeted Bacterial Control

Mateusz Wdowiak^1,*^, Aneta Magiera^1^, Magdalena Tomczyńska^1^, Witold Adamkiewicz^1^, Francesco Stellacci^2^, Jan Paczesny^1,*^

^1^ Institute of Physical Chemistry PAS, Marcina Kasprzaka 44/52, 01-224 Warsaw, Poland

^2^ École Polytechnique Fédérale de Lausanne, Station 12, CH-1015 Lausanne, Switzerland

^*^ corresponding author

KEYWORDS: bacteriophages, food dyes, stabilization, antimicrobial combinations, food preservation

**Additional information about protocols and methods**

***Instrumentation***

UV-Vis spectra were obtained using an Evolution 220 UV-visible spectrophotometer (Thermo Scientific, Waltham, Massachusetts, USA). Measurements were performed in quartz cuvettes (10 × 10 mm, Hellma). Wavelengths from 200 nm to 800 nm were examined with increments of 1 nm. Samples were adequately diluted if necessary, and the dilution factor was taken into account during the analysis.

***Comparison*** ***of the UV-protective properties of food dyes to commonly used staining dyes***

To verify that the protective properties of the food dyes were related to protein binding, we incubated T4 bacteriophage (10^5^ PFU/mL) in the 0.5% solutions (in TM buffer) of non-protein binding staining dyes – rhodamine B (used for cell membranes staining), eosin Y and SYBR green (used for DNA staining), and crystal violet (used for peptidoglycan staining). 0.5% Congo red solution was used as a control for protein-binding dye, and quinine solution (10 μg/mL, equal to the concentration of quinine in tonic water soft drink) was used to investigate the effects of UV-absorbing compound used in the food industry, that is not a colorant. The molecular structures of these compounds are given in **Scheme 1**. As a control, phages incubated in the TM buffer solution were used. After the overnight incubation in the examined solutions, T4 bacteriophages were titrated using the droplet method, then exposed to UV irradiation (CL-1000 Ultraviolet Crosslinker, UVP; 5 × 8 W, 254 nm UV) for 1 minute, and again titrated on the double-layer agar plates. The experiment was performed in triplicate. The results are presented in **Figure 1a** (main text).

**
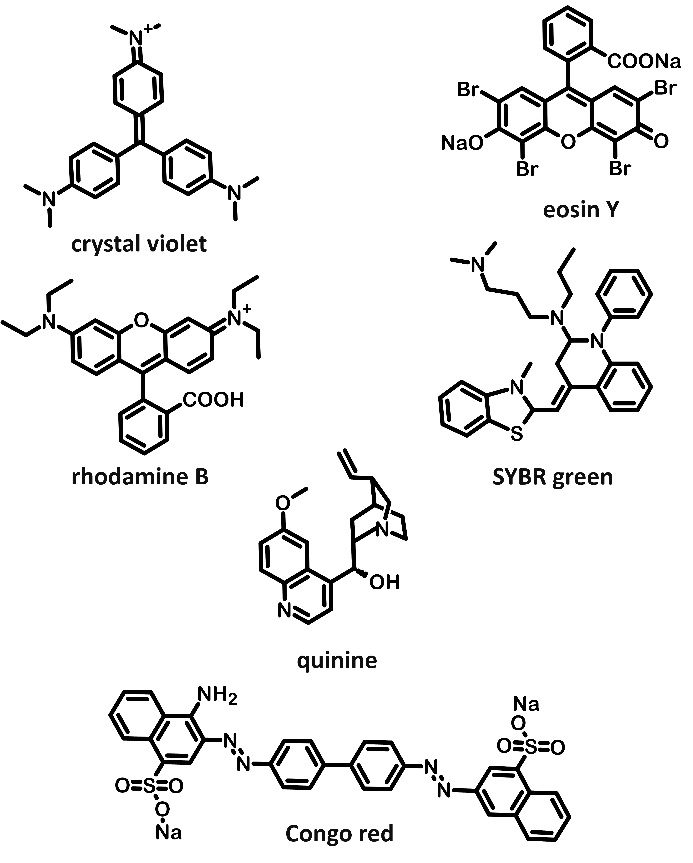
**

**Scheme 1**. Molecular structures of tested control compounds - rhodamine B (used for cell membranes staining), eosin Y and SYBR green (used for DNA staining), crystal violet (used for peptidoglycan staining), Congo red (protein-binding dye) and quinine (strong UV absorption).

**Additional results and explanations**

***Estimation of EC_50_ values***

The effective dose of protecting agents against UV irradiation was verified for four bacteriophages: T4, MS2, M13, and P22. Values of EC_50_, i.e., the concentration that provides 50% protection, were estimated using the Hill equation. The decrease of titers was evaluated for concentrations varying from 0.001% to 2%. Experiments were performed according to the protocol described in the *Bacteriophage protection against UV radiation and temperature* section in the main text. All the experiments were performed in triplicate. The results are presented in **Figures S1-S8** as the concentration (w/v).

The EC_50_ values of the examined food dyes were as follows:

- phage T4 - 0.11% for TR, 0.14% for QY, 0.15% for SY, 0.18% for PC, 0.20% for AR, 0.30% for AZ, 0.32% for BB, and 0.33% for IC,
- phage MS2 – 0.21% for TR, 0.14% for QY, 0.13% for SY, 0.19% for PC, 0.15% for AR, 0.28% for AZ, 0.42% for BB, and 0.32% for IC,
- phage M13 – 0.19% for TR, 0.14% for QY, 0.14% for SY, 0.21% for PC, 0.18% for AR, 0.32% for AZ, 0.41% for BB, and 0.40% for IC,
- phage P22 – 0.12% for TR, 0.15% for QY, 0.15% for SY, 0.18% for PC, 0.20% for AR, 0.29% for AZ, 0.32% for BB, and 0.33% for IC.

It is worth noticing that EC_50_ values for any given dye were similar for all phages. The EC_50_ values were significantly lower for all tested food dyes than Congo red (EC_50_ values in the 0.7% - 1.1% [1]). The food dyes provide protection similar to Congo red, but at lower concentrations, and are generally recognized as harmless for the environment and animals (including humans), allowing for the simultaneous application of phages and UV irradiation for bacteria eradication.

**
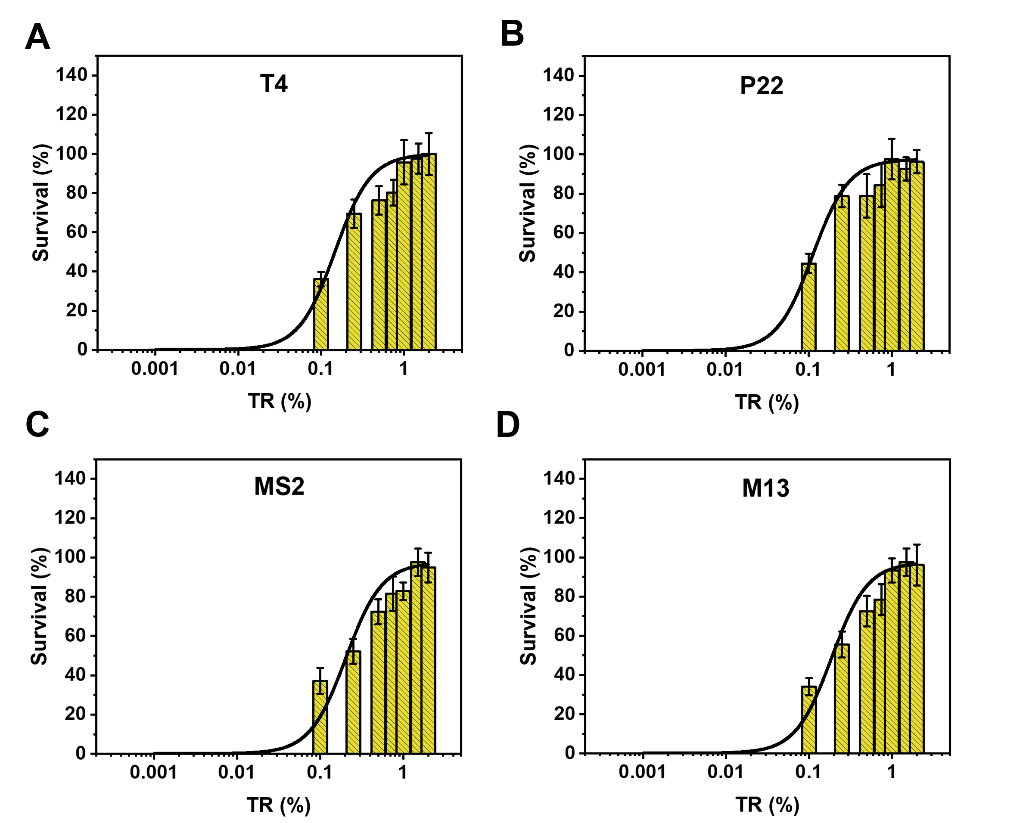
**

**
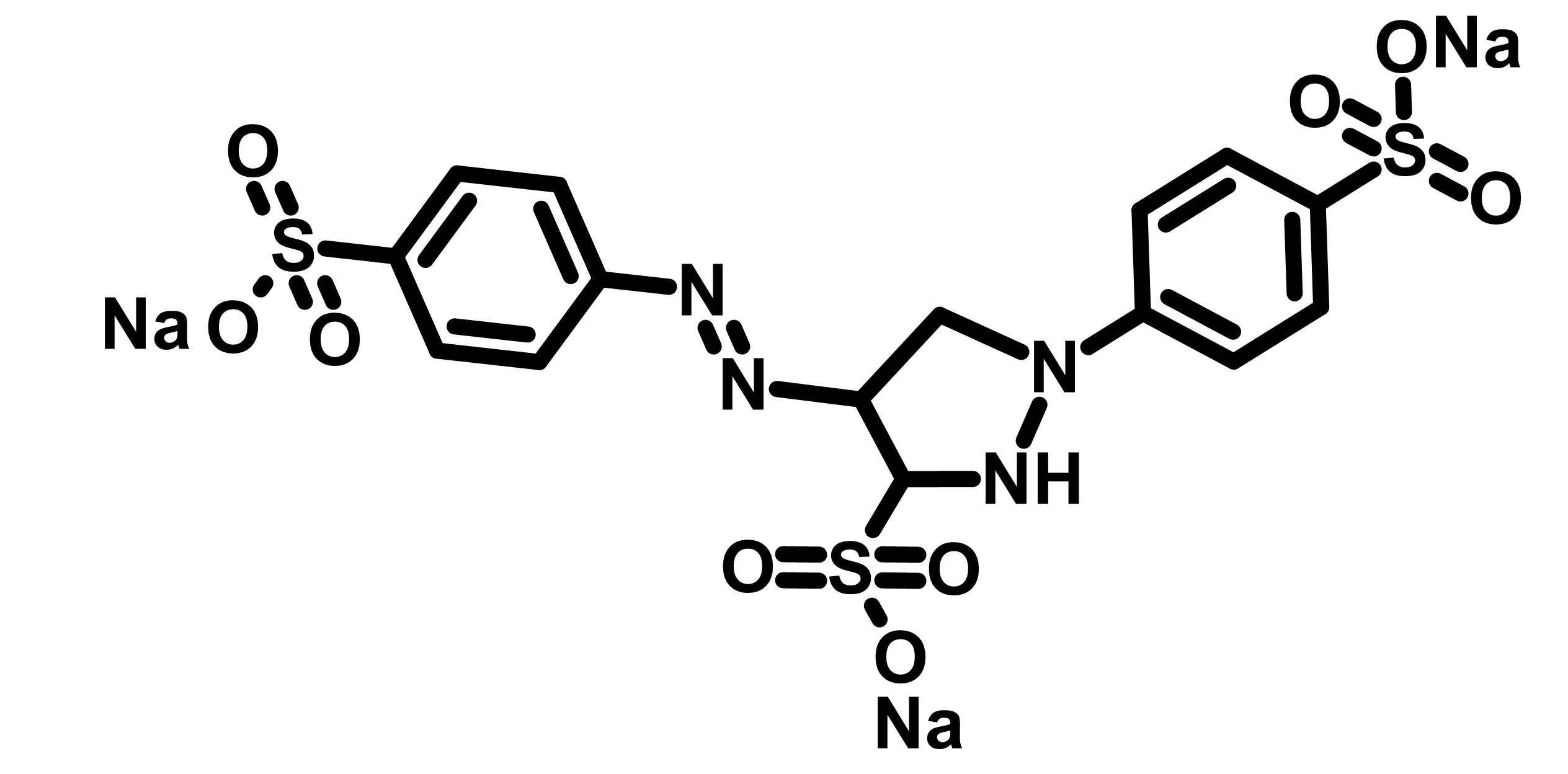
**

**Figure S1.** The estimation of EC_50_ values provided by tartrazine (TR) for **A)** T4 bacteriophage, **B)** P22 bacteriophage, **C)** MS2 bacteriophage, **D)** M13 bacteriophage.


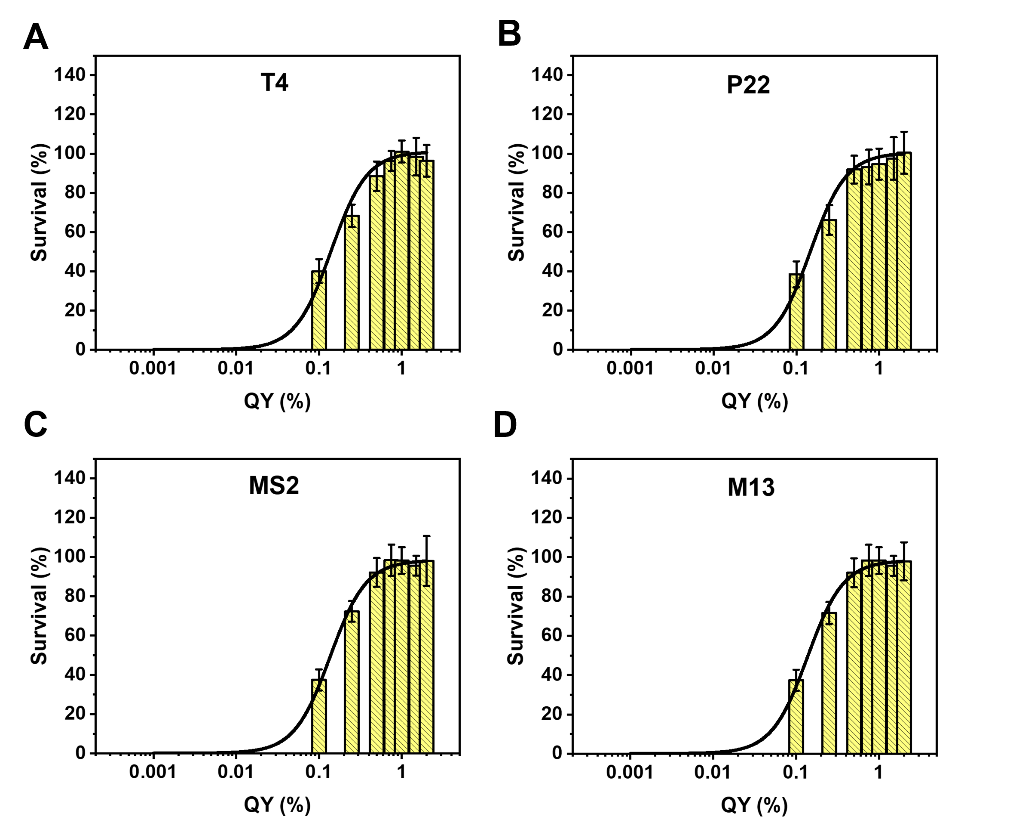


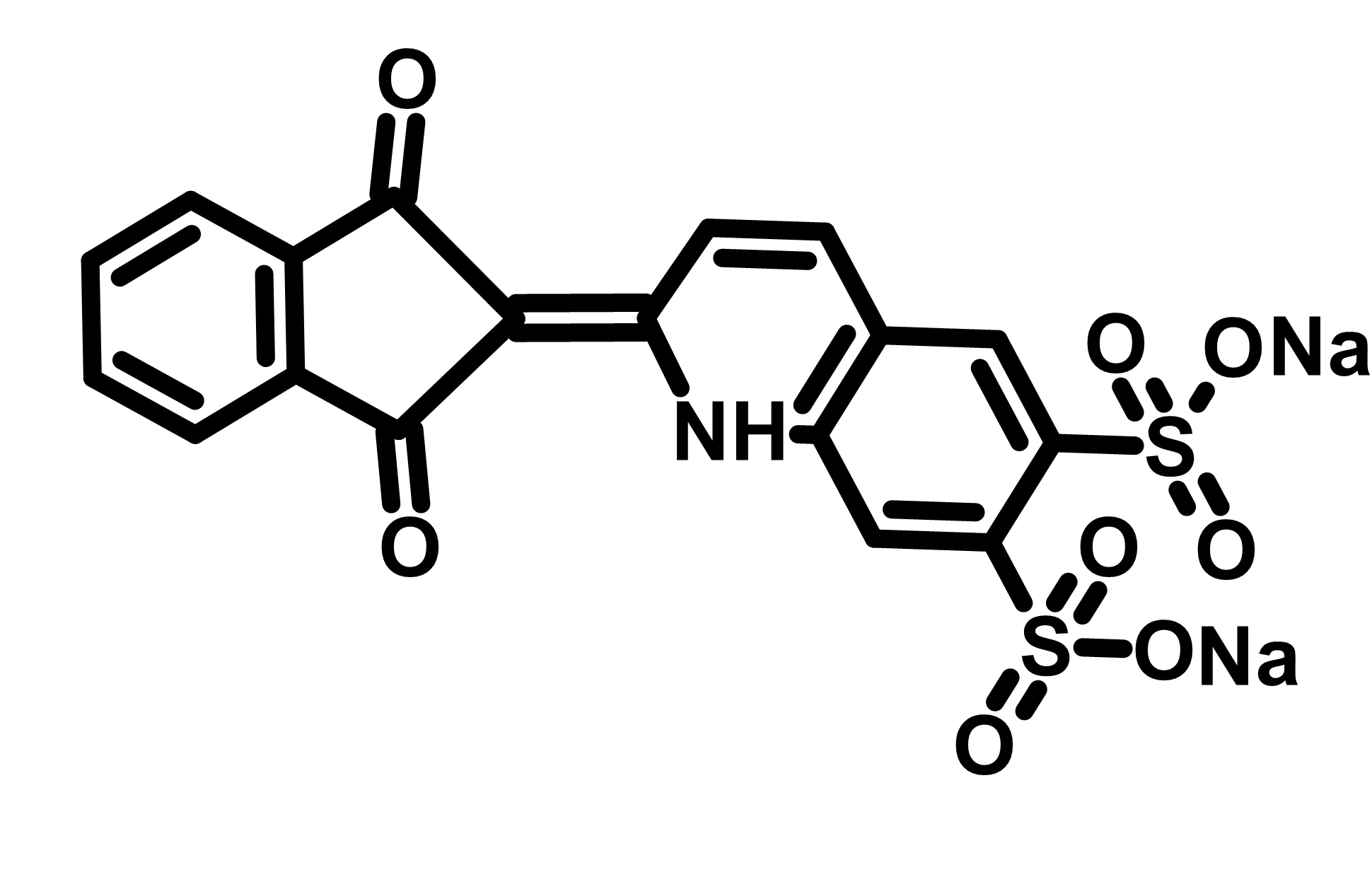


**Figure S2.** The estimation of EC_50_ values provided by quinoline yellow (QY) for **A)** T4 bacteriophage, **B)** P22 bacteriophage, **C)** MS2 bacteriophage, **D)** M13 bacteriophage.

**
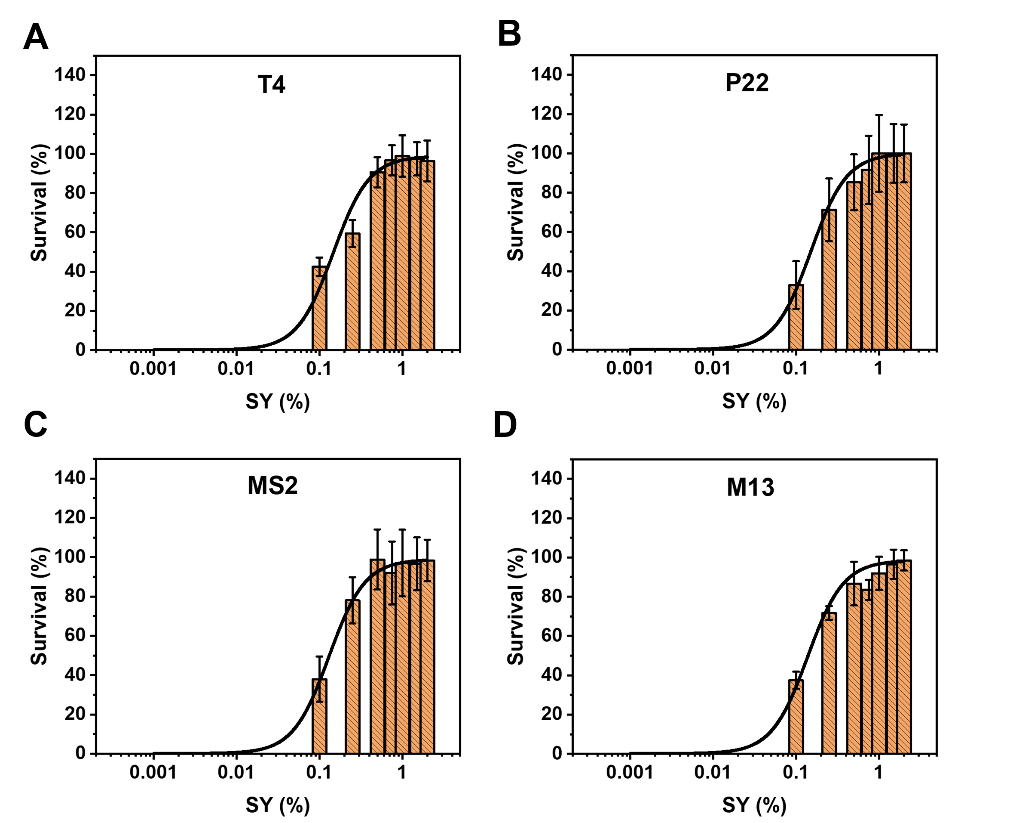
**

**
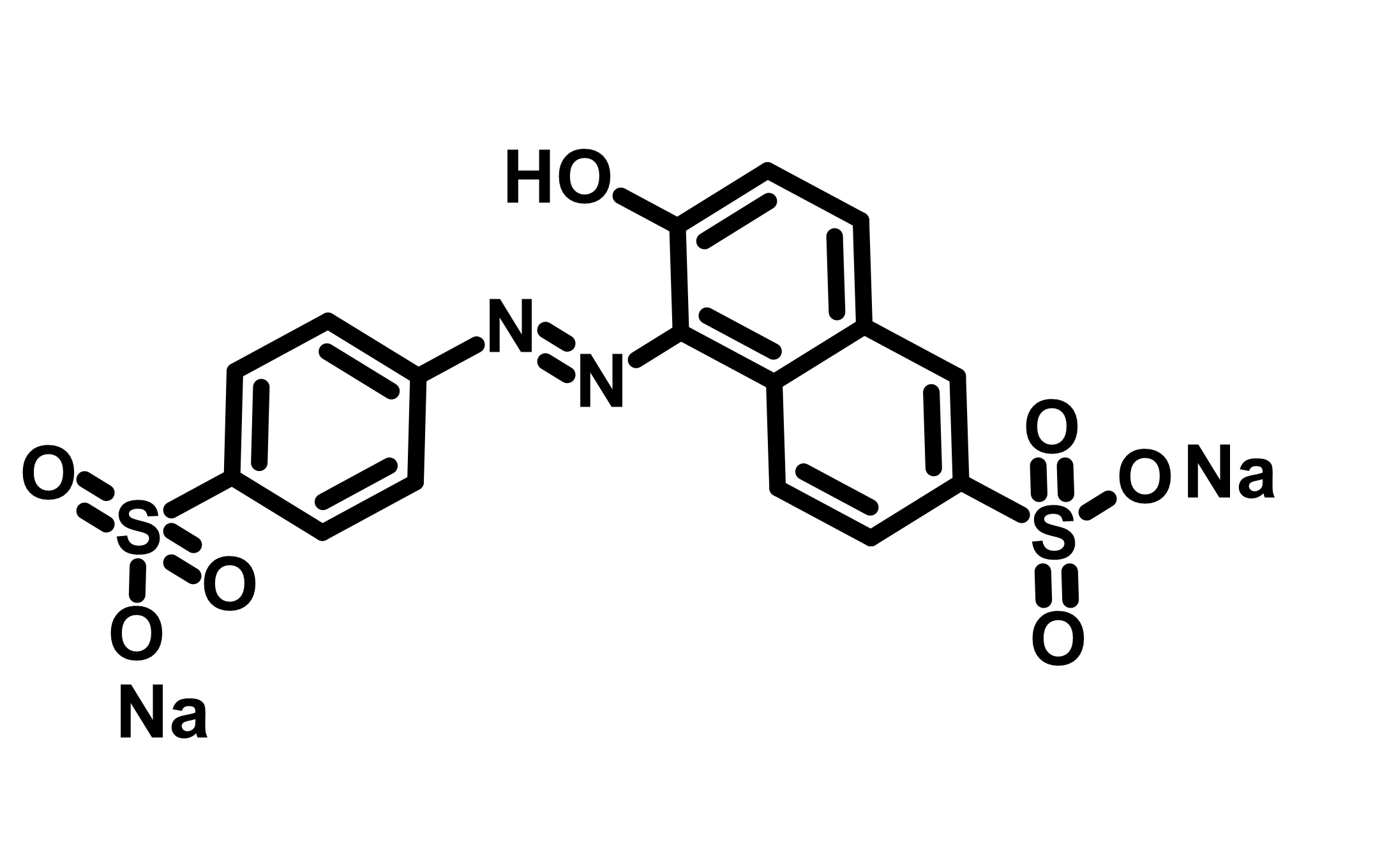
**

**Figure S3.** The estimation of EC_50_ values provided by sunset yellow FCF (SY) for **A)** T4 bacteriophage, **B)** P22 bacteriophage, **C)** MS2 bacteriophage, **D)** M13 bacteriophage.

**
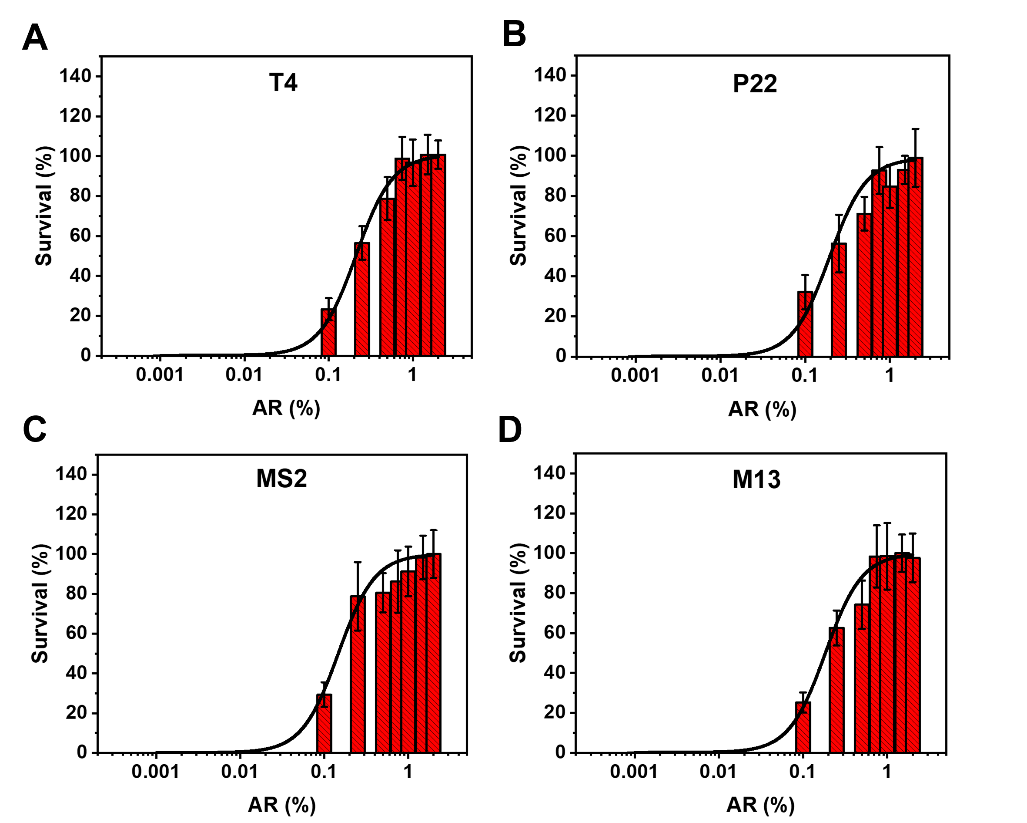
**

**
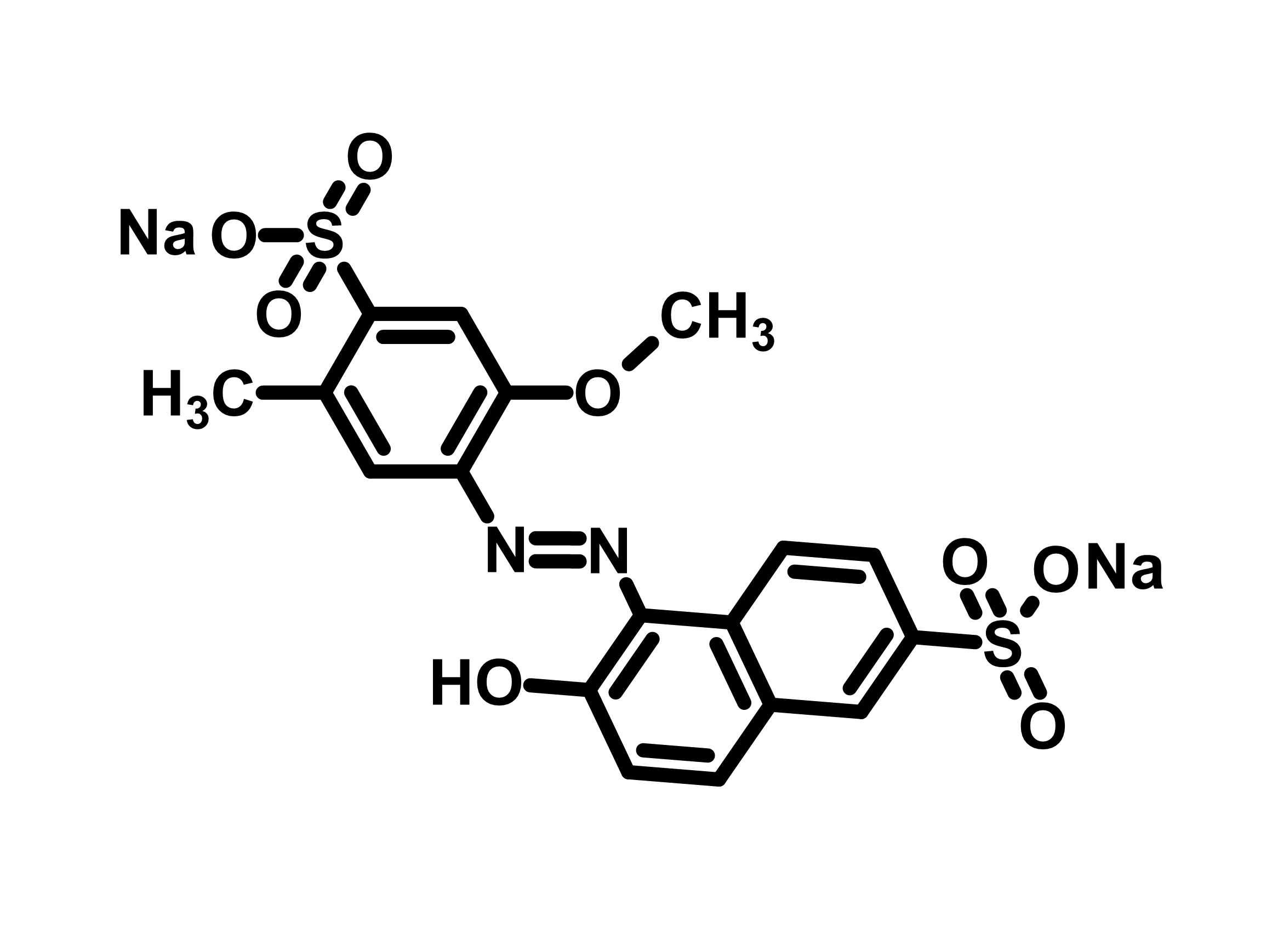
**

**Figure S4.** The estimation of EC_50_ values provided by Allura red (AR) for **A)** T4 bacteriophage, **B)** P22 bacteriophage, **C)** MS2 bacteriophage, **D)** M13 bacteriophage.

**
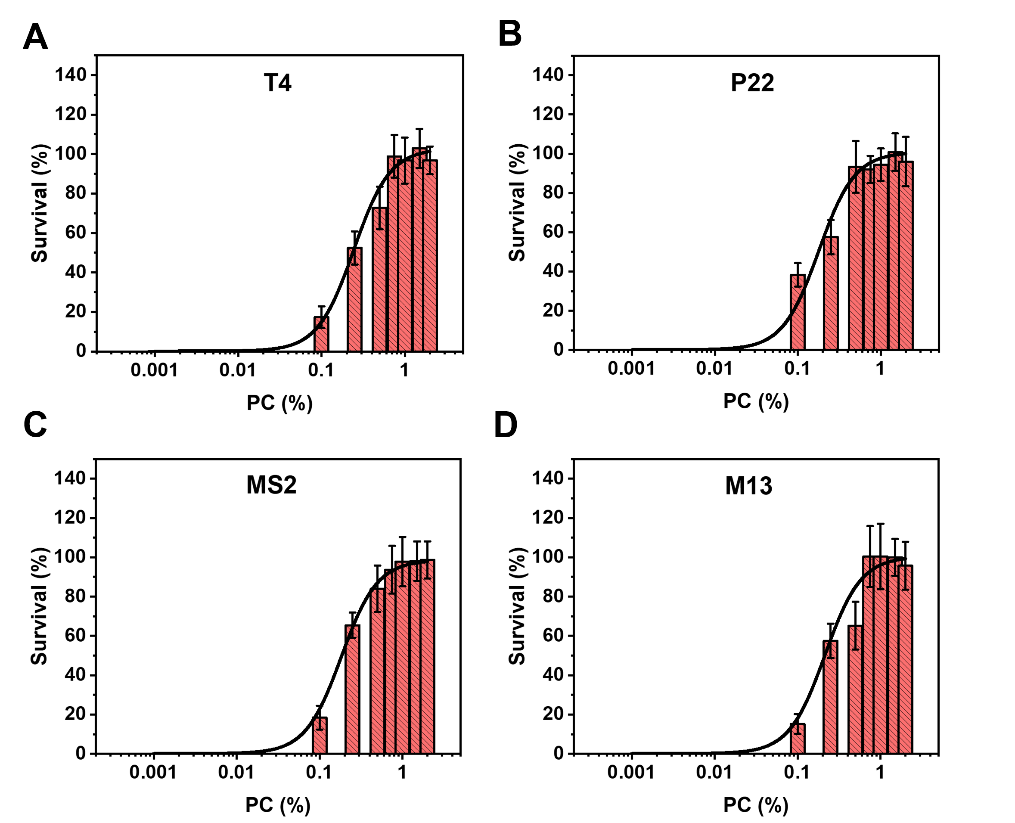
**

**
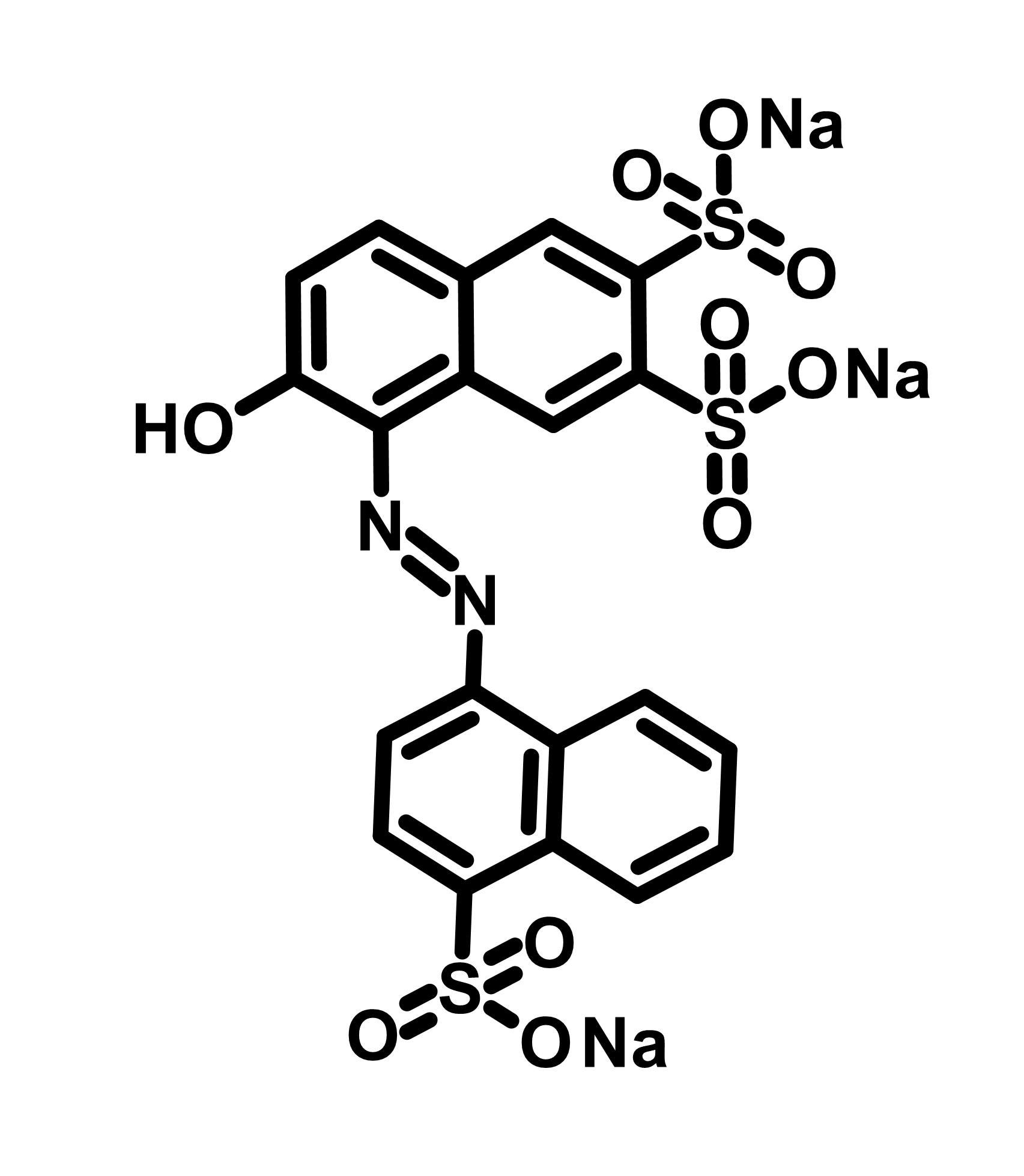
**

**Figure S5.** The estimation of EC_50_ values provided by Ponceau 4R (PC) for **A)** T4 bacteriophage, **B)** P22 bacteriophage, **C)** MS2 bacteriophage, **D)** M13 bacteriophage.

**
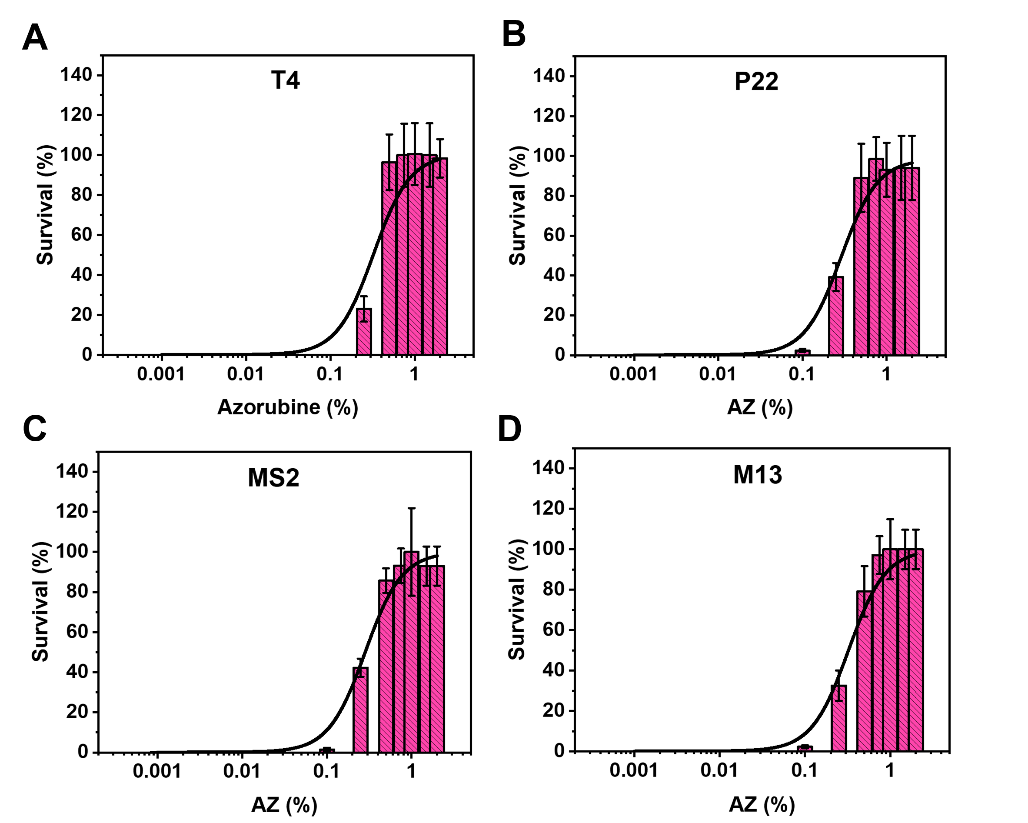
**

**
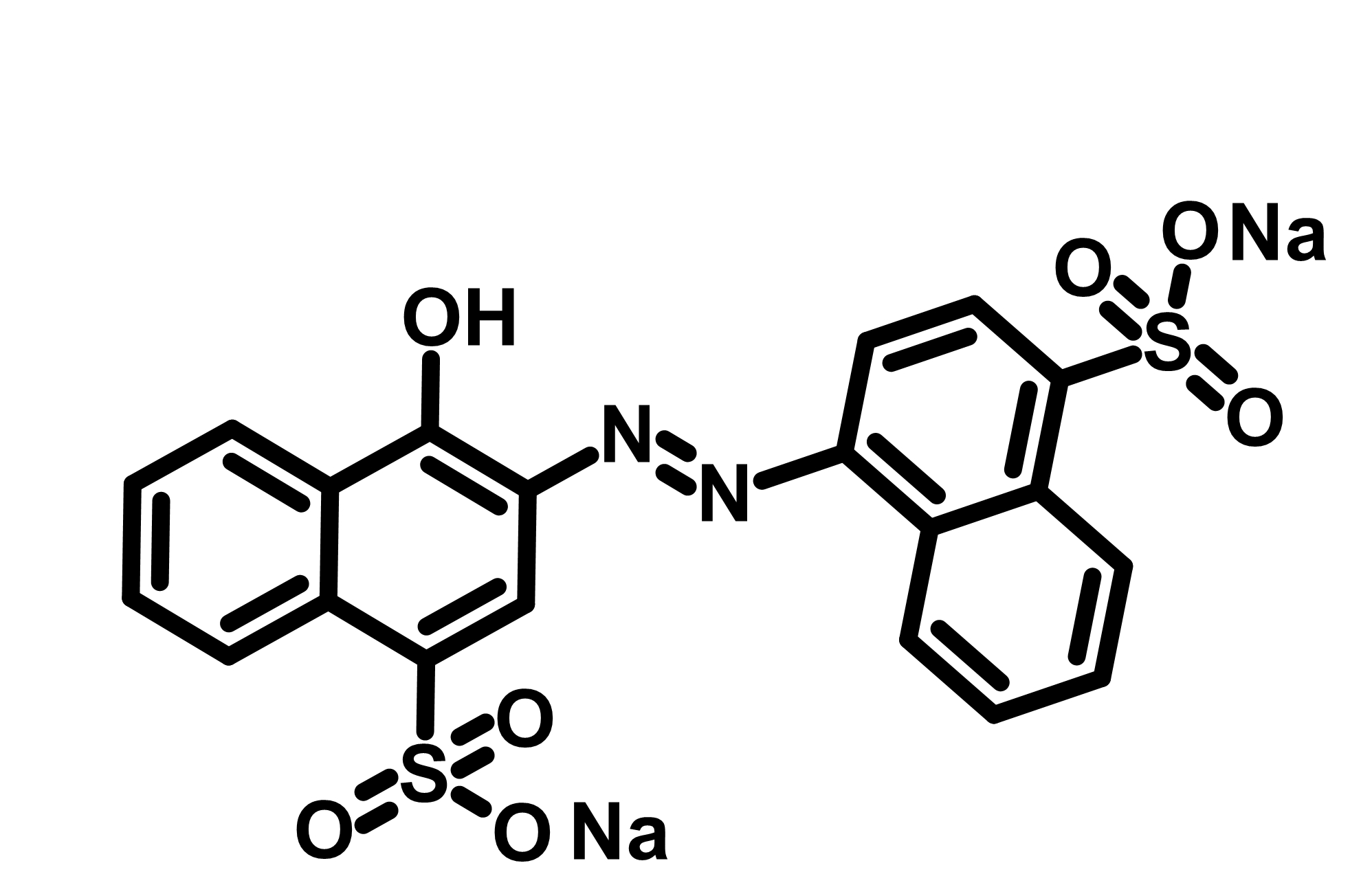
**

**Figure S6.** The estimation of EC_50_ values provided by azorubine (AZ) for **A)** T4 bacteriophage, **B)** P22 bacteriophage, **C)** MS2 bacteriophage, **D)** M13 bacteriophage.

**
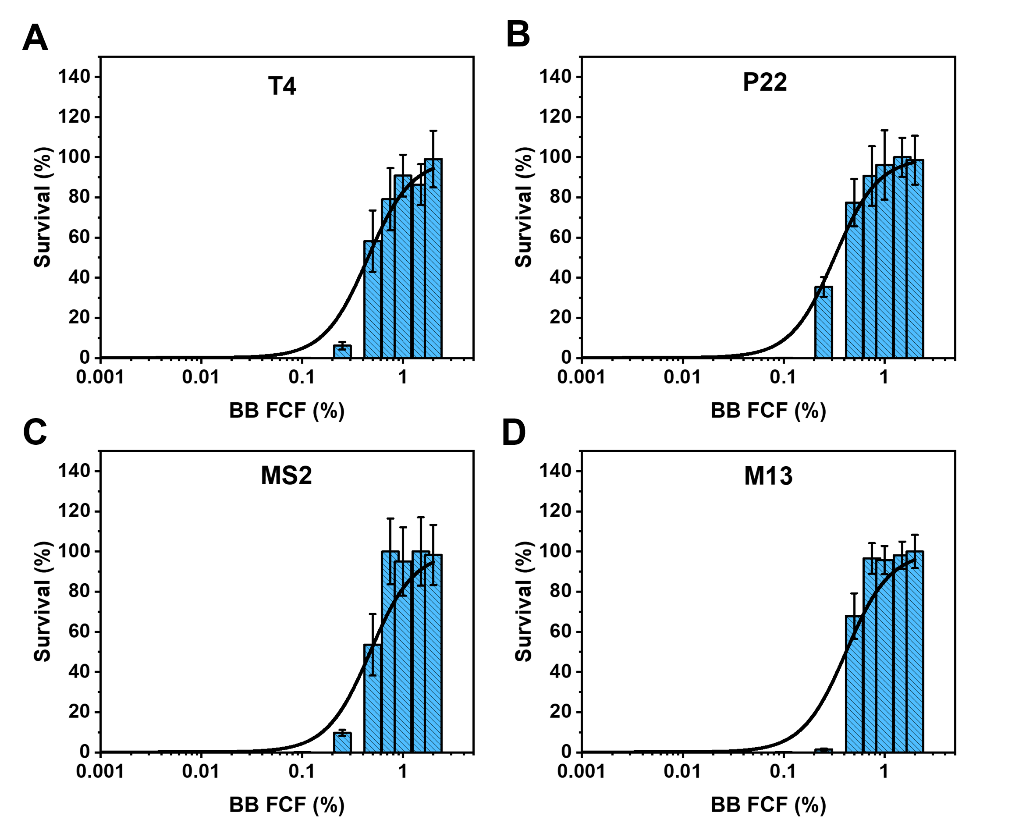
**

**
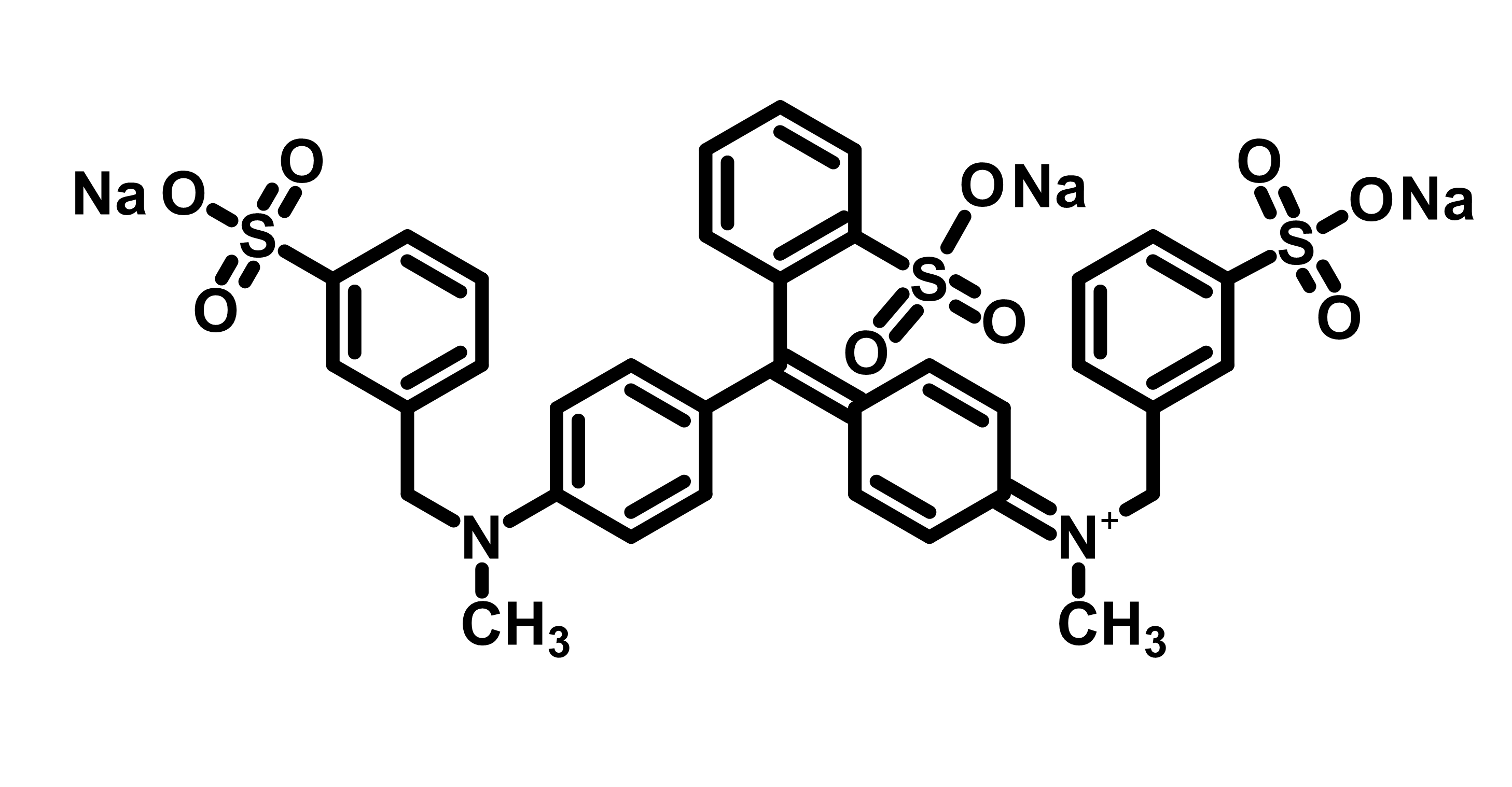
**

**Figure S7.** The estimation of EC_50_ values provided by brilliant blue FCF (BB) for **A)** T4 bacteriophage, **B)** P22 bacteriophage, **C)** MS2 bacteriophage, **D)** M13 bacteriophage.

**
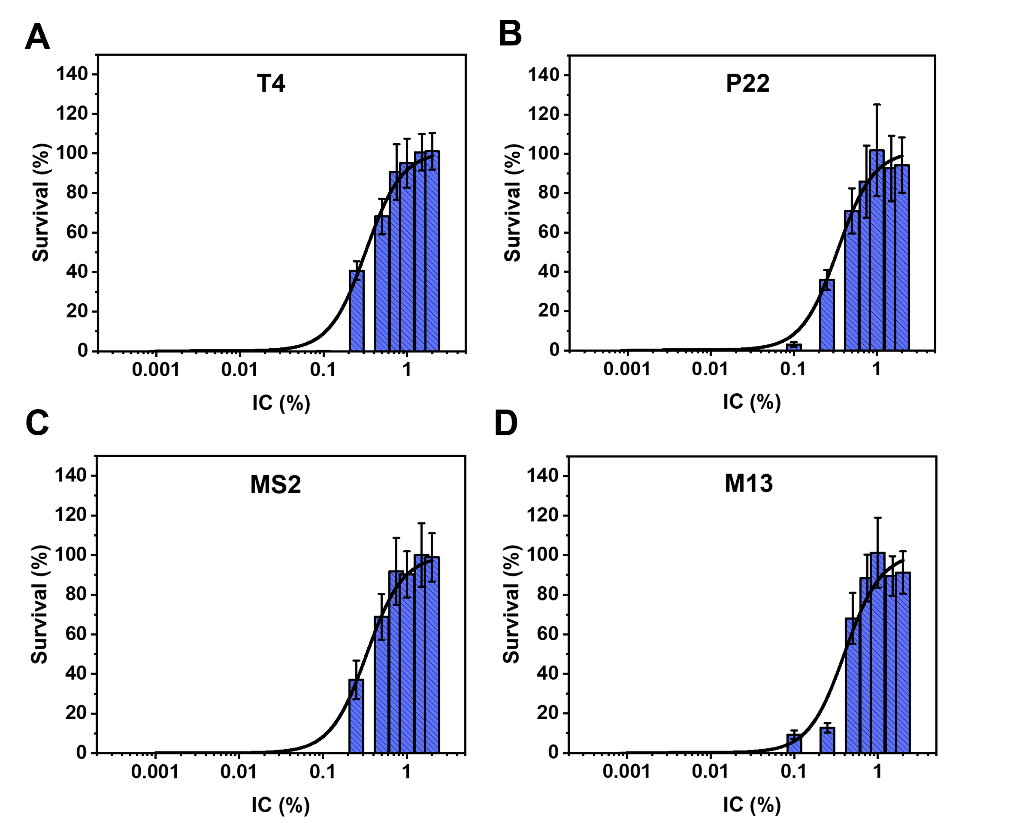
**

**
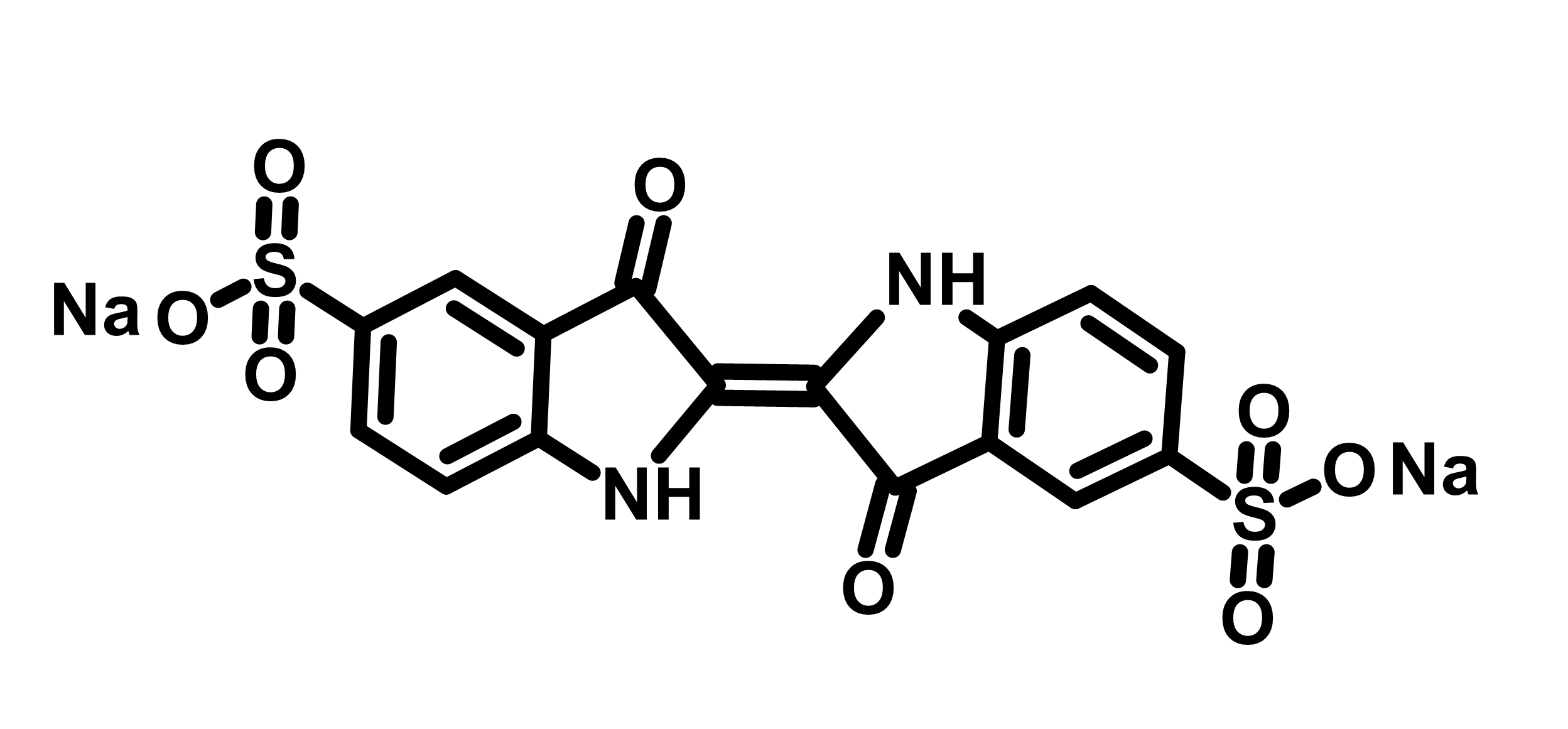
**

**Figure S8.** The estimation of EC_50_ values provided by indigo carmine (IC) for **A)** T4 bacteriophage, **B)** P22 bacteriophage, **C)** MS2 bacteriophage, **D)** M13 bacteriophage.

***Crystal violet and brilliant blue FCF comparison***

We compared BB with crystal violet (CV) – a triarylmethane dye commonly used to stain bacterial cell walls. Crystal violet was selected due to the similarities in molecular structure and the absorption spectrum in UV and visible light. T4 bacteriophages, *E. coli* BL21, and *S. aureus* ATCC 43300 strains were tested as models. The experiments were performed according to the protocol described in the *Materials and Methods* section in the main manuscript text. The UV-Vis spectra analysis was performed using an Evolution 220 UV-visible spectrophotometer (Thermo Scientific, Waltham, Massachusetts, USA). Measurements were performed in quartz cuvettes (10 × 10 mm, Hellma); during all measurements, wavelengths from 200 nm to 800 nm were examined, with increments of 1 nm.

BB protected T4 phages and *S. aureus* (Gram-positive) while remaining neutral to *E. coli* (Gram-negative bacteria). CV didn’t protect any tested samples, i.e., neither T4 phages nor examined bacteria survived UV irradiation (**Figure 1a** and **Figure S9**).


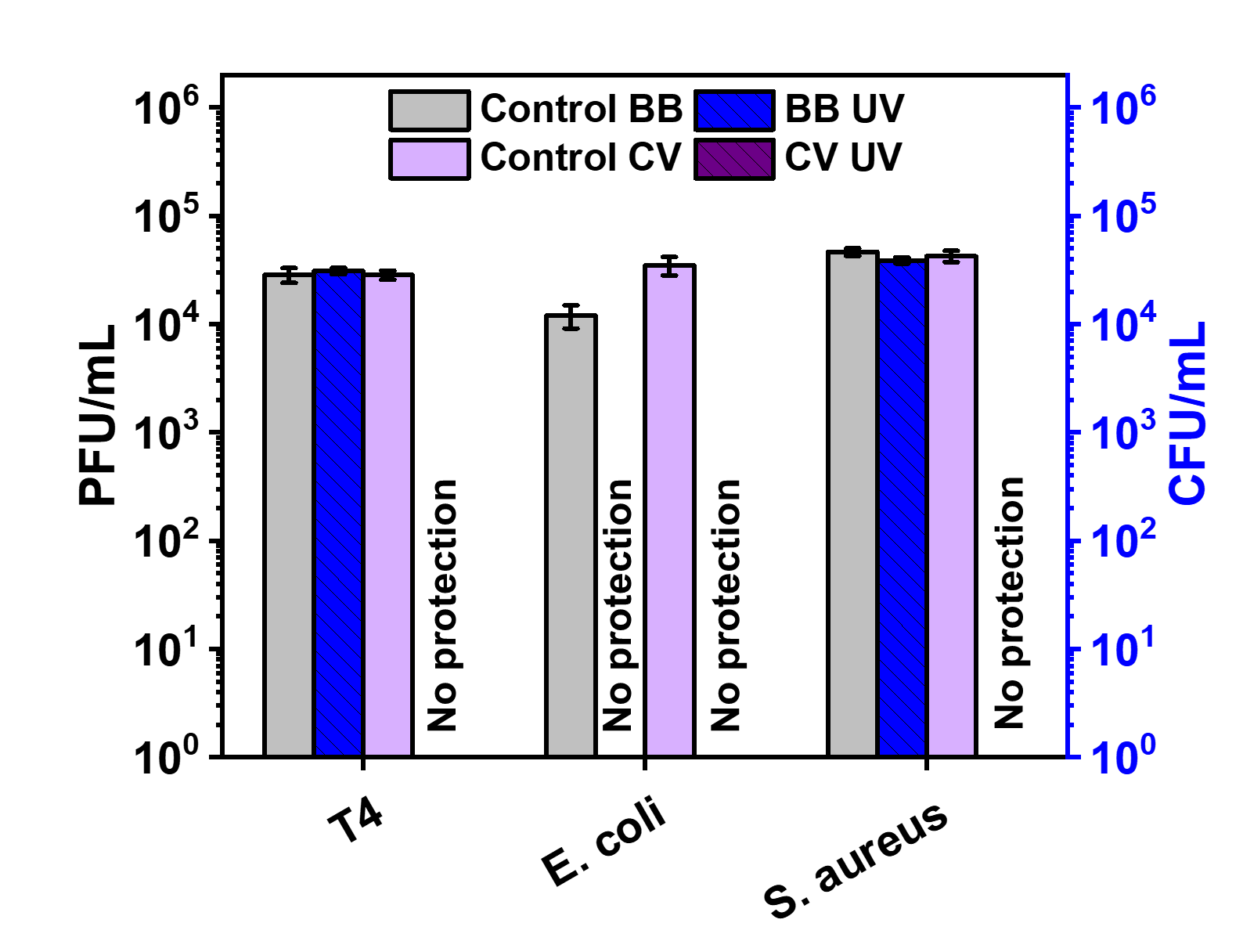


**Figure S9.** The comparison of UV-Vis spectra of BB and CV (left) and the evaluation of phage/bacteria-protective properties of CV compared to BB (right).

Both dyes possess a triarylmethane domain in the center of their molecular structures (**Scheme 1**). Besides the presence of additional aromatic rings in the structure of BB, the significant difference in the molecular structure of BB, compared to CV, is the presence of three sulphonic groups (SO_3_^-^). Sulphonic groups are described as crucial for protein-dye interactions. Their negative charge allows for the binding to positively charged amine groups in the side chains (NH_3_^+^) of basic amino acids, such as lysine, histidine, and arginine [2]. These interactions are the basis of the protein staining in SDS-PAGE gels using the derivative of brilliant blue FCF – Coomassie brilliant blue R250 and Coomassie brilliant blue G250 [2].

The UV-Vis spectra analysis revealed that both dyes have comparable absorbance in the UV spectrum, particularly for wavelengths of 210 nm and 300 nm. CV also presented an absorption peak at about 260 nm. Both dyes have the maximum absorbance in the spectrum at longer wavelengths: 590 nm for CV and 620 nm for BB (**Figure S9**).

***Small molecules as protectants***

We compared protective properties of sulfonic compounds of different molecular structures: sodium dodecyl sulfate (SDS; Sigma-Aldrich, Saint Louis, Missouri, USA), 2-(N-morpholino)ethanesulfonic acid (MES, Sigma-Aldrich, Saint Louis, Missouri, USA), toluene sulfonic acid (TSA; POCH, Gliwice, Poland), 4-hydroxybenzenesulfonic acid (HBSA; TCI Europe N.V., Zwijndrecht, Belgium), sodium 2-naphthol-6-sulfonate (NSA; TCI Europe N.V., Zwijndrecht, Belgium), and sodium isatin-5-sulfonate (ISA; Sigma-Aldrich, Saint Louis, Missouri, USA). As a negative control to sodium isatin-5-sulfonate, isatin (Sigma-Aldrich, Saint Louis, Missouri, USA) was used. Isatin was a derivative of and isatin-5-sulfonate devoid of the sulfonic group (SO_3_^-^). Before the experiment, the acidic compounds (toluene sulfonic acid and 4-hydroxybenzene sulfonic acid) were neutralized using 0.1M NaOH solution.

The UV-Vis spectra analysis revealed that SDS and MES have almost no absorbance for the radiation from the UV and visible radiation. Other compounds showed some absorbance in the UV region, particularly at 220 nm and/or 250 nm. HBSA, NSA, and ISA also showed an absorbance peak at the 300 nm wavelength (**Figure S10**). All the examined aromatic compounds effectively absorbed UV irradiation.


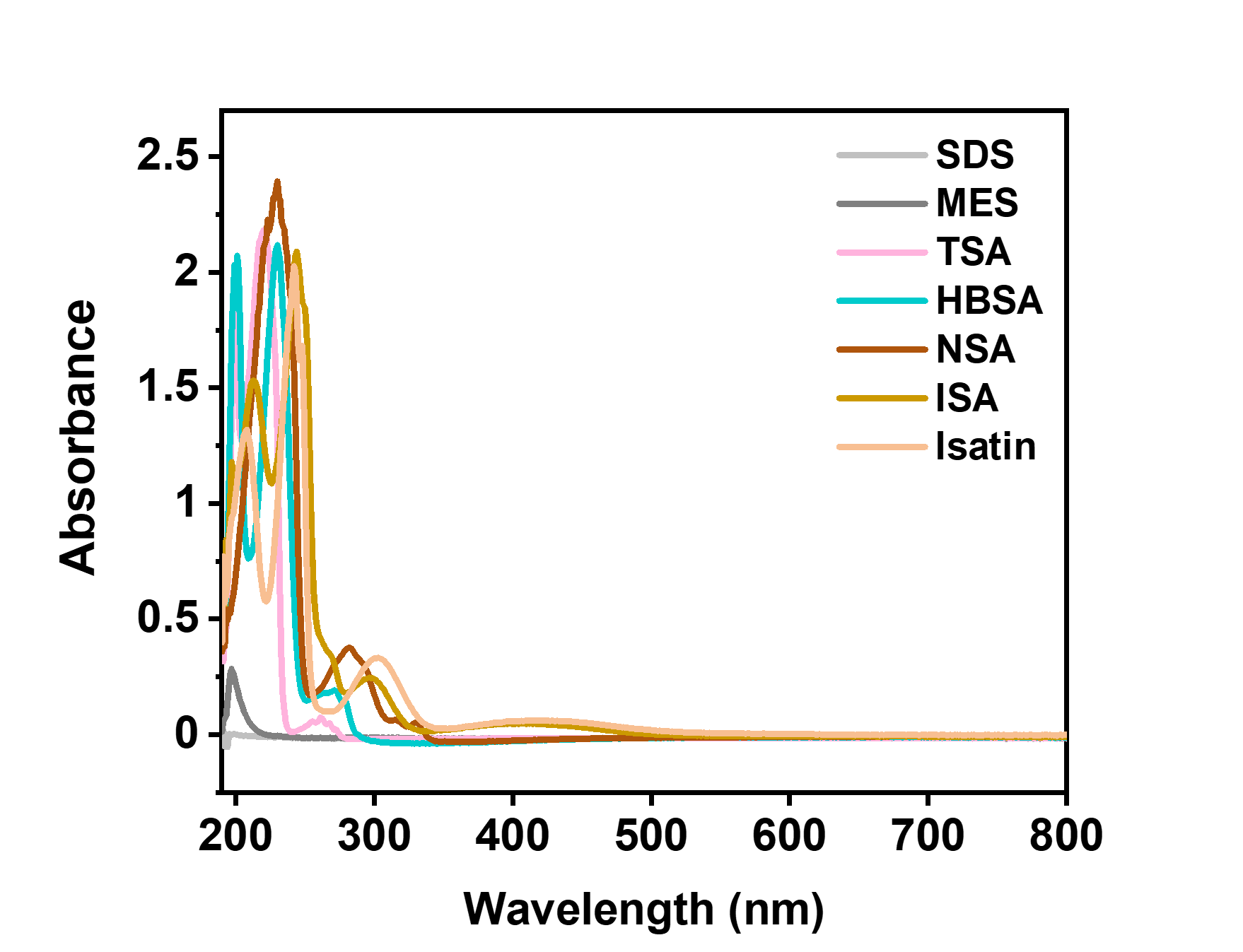


**Figure S10.** The UV-Vis spectra of sodium dodecyl sulfate (SDS), 2-(N-morpholino)ethanesulfonic acid (MES), toluene sulfonic acid (TSA), 4-hydroxybenzenesulfonic acid (HBSA), sodium 2-naphthol-6-sulfonate (NSA), and sodium isatin-5-sulfonate (ISA), and sodium isatin-5-sulfonate (isatin).

Simultaneously, with minor differences compared to the effects of bacteriophages, NSA and ISA also protected Gram-negative (*E. coli* BL21) and Gram-positive (*S. aureus* ATCC 43300) (**Figure 3b** and **Figure S11**). After 1 minute of UV irradiation, the titer of bacteria incubated in ISA solution was similar to before the exposure. This rate was similar to the stabilization rate of indigo carmine. For NSA, the bacterial count after the UV exposure dropped slightly. Surprisingly, HBSA turned out to be toxic to *E. coli* (**Figure 3b**) and *S. aureus* (**Figure S11**), reducing bacterial titer to below 10 CFU/mL upon incubation but without UV. Isatin presented moderate antibacterial properties, reducing bacterial titer by about 1-log. A further reduction by an additional 1-log was observed after the UV irradiation. Therefore, isatin possessed moderately antibacterial activity (which was in line with the current state of the knowledge [3]) and moderately protective properties.


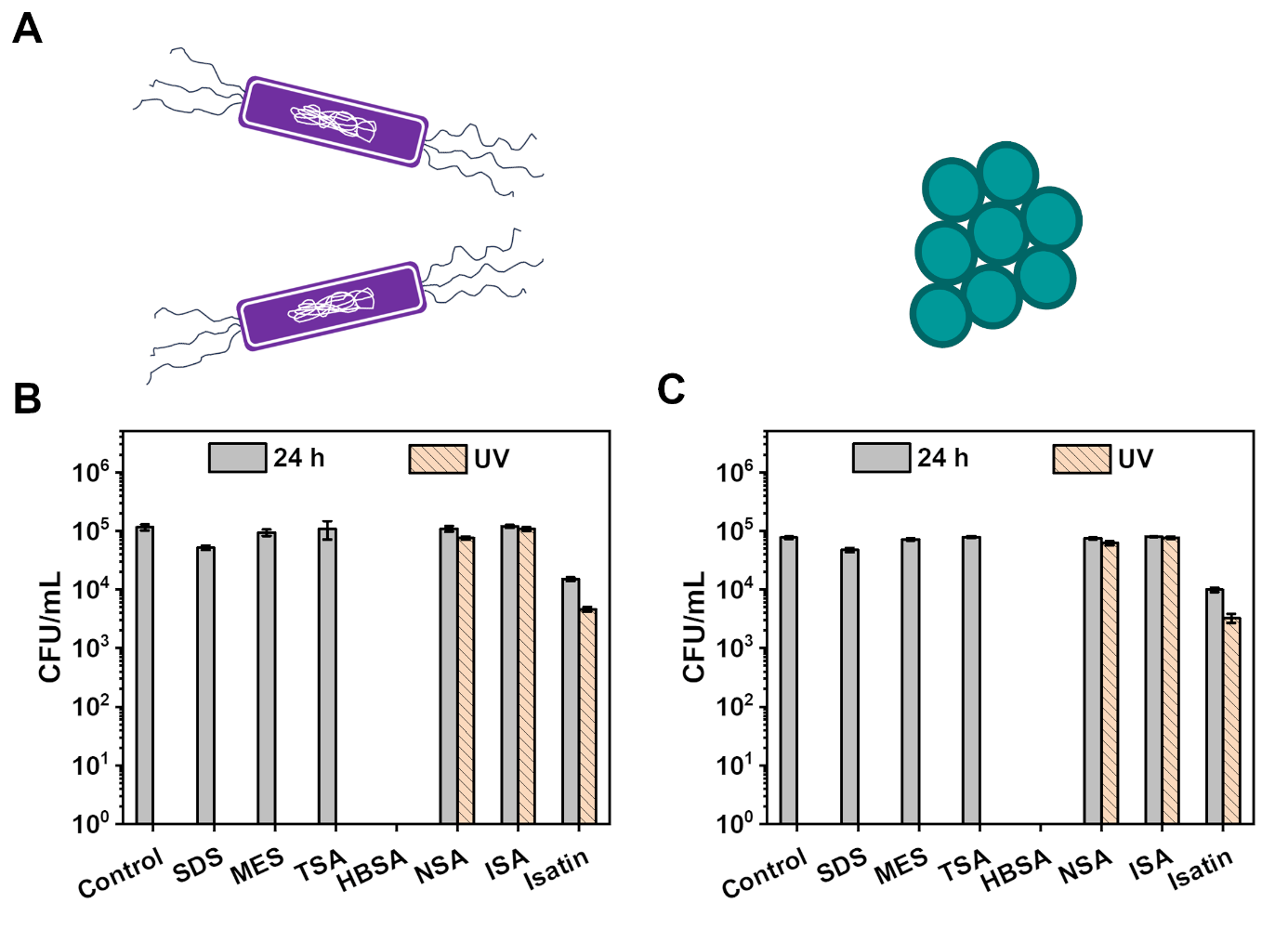


Figure S11. The protective properties of the tested small molecules against *S. aureus* ATCC 43000 were examined. Results for *E. coli* BL21 (Gram-negative) are presented in the main text (Figure 3c).

***Components of selected phages and bacteria***

The comparison of the structures of major outer proteins of bacteriophages and bacteria included in this study suggested that out membrane proteins of some Gram-negative bacteria contained less basic amino acids, compared to phage and Gram-positive bacteria proteins. Based on the kinetics of BB interactions with proteins, this may hint at the explanation of the selectivity of BB and Congo red and open new frontiers for the research on protein and virus interaction with small molecules. The analysis was based on structures of T4 bacteriophage major capsid protein gp23 (P04535 (CAPSH_BPT4)), *Salmonella* P22 phage major capsid protein (P26747 (CAPSD_BPP22)), *Escherichia* phage MS2 capsid protein (J9QBW2 (J9QBW2_BPMS2)), Enterobacteria phage M13 capsid protein G8P (J7I0P6 (J7I0P6_BPM13)), *E. coli* outer membrane protein A (P0A910 (OMPA_ECOLI)), and *S. aureus* surface protein G, A-domain (Q2G2B2 (SASG_STAA8)), with their contents of basic amino acids. The structures of the proteins were acquired from the Swiss-Model Expasy database.

***Cytotoxicity of the brilliant blue FCF***

Cytotoxicity tests were performed using MTT proliferation/metabolic activity assays. We used two different cancer cell lines: HeLa (cervical cancer) and A549 (lung cancer). The MTT assay was performed using around 10,000 cells/well for both tested cell lines (controlled with Countess II Cell Counter). Cells were seeded into a 96-well plate (Greiner Bio-One) and incubated in an incubator for 6 hours at 37°C. Then, the medium was removed, and the tested formula (containing dye and polymer) at five different concentrations (double dilutions) was added to the fresh cell medium. The experiments were repeated five times for each concentration. After 6 hours of incubation, the medium was replaced with a culture medium including 1 mM 3-(4,5-dimethylthiazol-2-yl)-2,5-diphenyltetrazolium bromide (MTT reagent, Thermo Fischer Scientific). Cells were incubated with MTT reagent for 3 hours at 37°C. Then, solutions were replaced with DMSO and incubated for 10 minutes. The absorbance in each well was measured at 540 nm using a SpectraMax i3x MultiMode Microplate Reader with injectors (Molecular Devices). For each experiment, negative and positive controls were included. Negative controls contained 1% Triton-X 100, while positive controls were cells cultured under standard conditions without the tested formula.

The MTT proliferation/metabolic activity test was performed using HeLa and A549 cell lines. After 6 hours of incubation in the BB solution, the viability of both HeLa and A549 cells was about 70-75%, i.e., within an acceptable range for biomedical protocols [4].

**Statistical analysis**

All the experiments were performed in triplicate. Unless stated otherwise, the statistical analysis against the control sample was performed using a Student t-test, * corresponding to p-value 0.05, ** to 0.01, *** to 0.001.

**Figure 4** in the main text shows the statistical differences between the ‘cleaning mixture’ solutions (BB-stabilized bacteriophages under UV exposure). This was to provide better clarity and readability of our results. The statistical significances between the remaining sample pairs are provided in the tables (**Table S1-S4**). The pairs between which the differences were statistically insignificant (p-value > 0.05) were marked as ‘ns’ (not significant). Non-correlated sample pairs are marked as ‘na’ (not applicable).

**Table S1.** Statistical analysis for the experimental results presented in **Figure 4** (membranes *E. coli*).

**Table S2.** Statistical analysis for the experimental results presented in **Figure 4** (membranes *B. subtilis*).

**Table S3.** Statistical analysis for the experimental results presented in **Figure 4** (food *E. coli*).

**Table S4.** Statistical analysis for the experimental results presented in **Figure 4** (food *S. enterica*).

**References**

[1] M. Wdowiak, P.A. Mierzejewski, R. Zbonikowski, B. Bończak, J. Paczesny, Congo red protects bacteriophages against UV irradiation and allows for the simultaneous use of phages and UV for membrane sterilization, Environ. Sci. Water Res. Technol. (2023). https://doi.org/10.1039/D2EW00913G.

[2] R.G.E. Krause, J.P.D. Goldring, Crystal violet stains proteins in SDS-PAGE gels and zymograms, Anal. Biochem. 566 (2019) 107–115.

[3] H. Guo, Isatin derivatives and their anti-bacterial activities, Eur. J. Med. Chem. 164 (2019) 678–688.

[4] J. Weyermann, D. Lochmann, A. Zimmer, A practical note on the use of cytotoxicity assays, Int. J. Pharm. 288 (2005) 369–376. https://doi.org/10.1016/j.ijpharm.2004.09.018.
